# Miniaturized HOXB13 Mimetics Are Sequence-Specific, Methyl-Sensitive DNA Binders

**DOI:** 10.1021/jacs.6c01290

**Published:** 2026-07-14

**Authors:** Claire Wong, Shiao Y. Chow

**Affiliations:** Department of Pure and Applied Chemistry, 3527 University of Strathclyde, G1 1XL Glasgow, U.K.

## Abstract

Homeobox protein Hox-B13 (HOXB13) is an oncogenic transcription factor that is associated with prostate cancer risk. Unlike most transcription factors (TFs), HOXB13 is a methyl-plus TF and has been shown to be preferentially recruited to DNA prostate cancer risk loci. Here, we miniaturized HOXB13 to create metal-stapled mimetics that can target its cognate primary DNA-binding site in a sequence-specific, methyl-sensitive manner. Targeted profiling of 5-methylcytosine (5mC) recognition using V269X mimetic mutants revealed that leucine can further enhance both methyl sensitivity and methyl specificity through methyl–methyl contact with 5mC and decreased methyl–pi interactions with cytosine. Collectively, our study revealed novel insights into the molecular interactions of HOXB13 with methylated DNA and new tools that will provide a structural basis for future development of a new class of sequence-specific methylated DNA binders.

## Graphical abstract

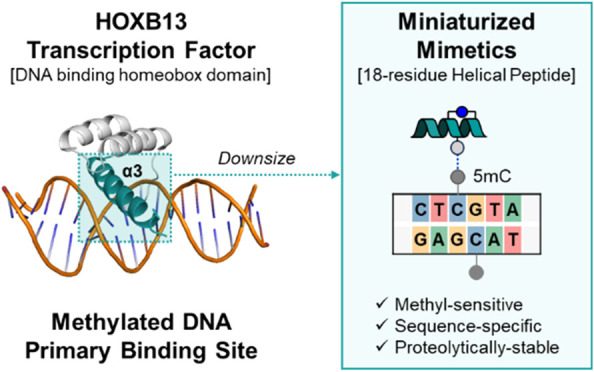

## Introduction

Transcription factors (TFs) are sequence-specific DNA binders that modulate gene expression. A single TF can bind multiple DNA-binding sites with varying binding affinities.^1^ Therefore, identifying TF binding preferences and how genomic features influence TF interactions is pivotal to advance our understanding of transcriptional regulation. Epigenetic modifications such as DNA methylation can alter TF-DNA interactions. 5-Methylcytosine (5mC) is read by methyl-CpG-binding domain (MBD) proteins in a sequence-independent manner (Figure 1). This is traditionally believed to impede TF binding to their cognate recognition motifs, thereby silencing gene expression.^2–4^ However, more recent evidence has suggested that TFs can also recognize methylated DNA in a sequence-specific manner.^5^ DNA methylation could either enhance or inhibit TF binding,^6^ leading to the discovery of various methyl-sensitive TFs and the identification of a new subset of ‘methyl-plus’ TFs (e.g., HOXB13 and OCT4 homeodomain proteins) with increased affinity for methylated DNA.^7^ Importantly, DNA methylation sites at certain genomic regions, such as downstream of transcription start sites,^8^ have been found to be positively correlated with oncogenic transcriptional activity.^8–10^ New tools for the interrogation of methylated DNA-TFs interactions are therefore urgently needed to bring critical insights to this important class of epigenetic markers to better understand disease progression at a genomic level.

**Figure 1:**
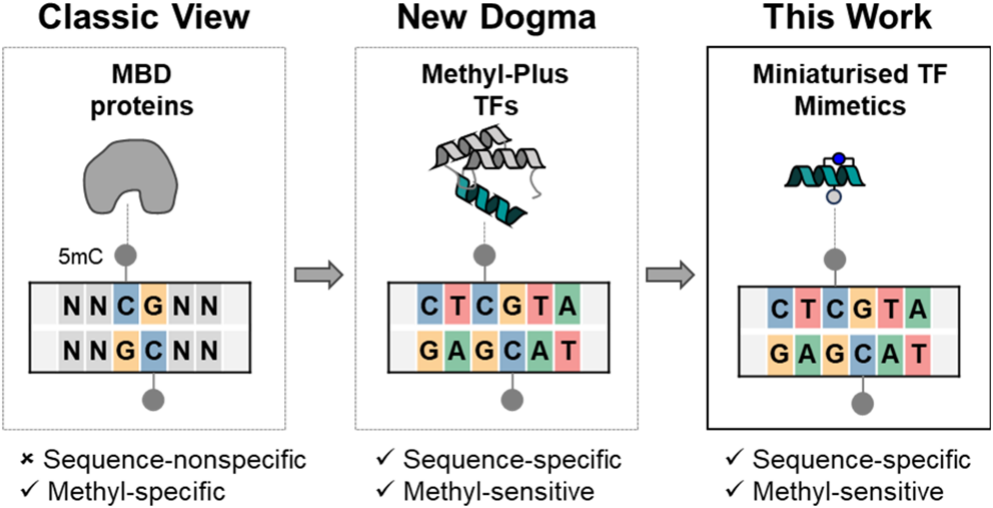
*Classic view*: MBD proteins can recognize methylated DNA species in a sequence-independent manner. *New dogma*: Methyl-plus TFs can recognize specific methylated DNA motifs. *This work*: Mimetics derived from HOXB13, a methyl-plus TF, are methyl-sensitive, sequence-specific DNA binders. C = cytosine; A = adenosine, *T* = thymine, G = guanine, 5mC = 5-methylcytosine, *N* = any base.

Despite the importance of DNA methylation in transcriptional regulation, significant knowledge gaps still exist. First, the governing rules of how methyl-plus TFs recognize their cognate methylated DNA are unclear, as most models for DNA-TF binding do not accommodate modified bases.^1,11^

Second, there is a lack of tools that can directly study specific methylated DNA motifs. Antibody-labeling technologies—derived from MBD proteins—can detect global methylated DNA population;^12^ however, they lack the prerequisite sequence specificity to detect biomarkers of interest at a locus-specific resolution. This necessitates the development of new tools to study methylated DNA biomarkers and their interactions in physiologically relevant contexts.

Miniaturization of transcription factors has emerged as an exciting strategy to study DNA biomarkers at sequence-specific resolution. Most protein DNA binders employ an α helix motif for sequence-specific DNA biorecognition.^13^ While DNA biorecognition motifs synthesized in isolation often lack the ability to bind DNA due to peptide unfolding, creating helical mimics of the biorecognition motif can reduce entropy penalty through structural preorganization to generate high-affinity sequence-specific DNA binders.^14^ Importantly, stapled peptides can further offer enhanced cellular penetration and proteolysis resistance due to conformational constraints.^15^ Several groups have demonstrated that stapled peptides derived from α-helical DNA recognition motifs of TFs can bind double-stranded DNA (dsDNA) in a sequence-specific manner,^14^ disrupt bacterial gene expression,^16^ and even enter live cancer cells.^17^ Combining the exquisite specificity of biologics and mutability of synthetic molecules, helical peptide mimics represent an exciting modality for programmable, fit-for-purpose DNA interrogation to study transcriptional regulation.

To date, there are only five reports on DNA-targeting stapled peptides that demonstrated sequence-specific disruption of DNA–protein interactions;^16–20^ however, none has been reported for methylated DNA. Here, we leveraged a methyl-sensitive transcription factor as a template to create first-in-class metallopeptides that recognize methylated DNA. Homeobox protein Hox-B13 (HOXB13) is a ‘methyl-plus’ transcription factor that strongly binds methylated DNA motif (CTmCGTAAA) over its unmethylated counterpart.^7,21^ Preferential recruitment of homeodomain protein HOXB13 to prostate cancer risk loci associated with elevated methylation has been reported to initiate oncogenic transcriptions.^22^ Although HOXB13 displays the strongest methyl-preference reported in the literature,^7^ the exact recognition basis remains unclear.

In this study, we create synthetic metallopeptides to target a cancer-associated methylated DNA motif and dissect the mechanism by which methylated DNA interacts with HOXB13, and we shed light on amino acid preference for methyl contact with 5-methylcytosine.

## Results

### Miniaturization Strategy of HOXB13 Protein

Inspired by reports of TF miniaturization for cognate DNA biorecognition, we investigated whether downsizing HOXB13 can create miniature methyl-sensitive, sequence-specific DNA binders that retain binding affinity to its cognate sequence. Composed of three α-helices, the DNA-binding homeobox domain of HOXB13 interacts with the major groove of DNA using the third α-helix (α3) and the minor groove using its *N*-terminus tail.^21^ Crystal structure of HOXB13 cocrystallized with its cognate methylated DNA motif revealed that the α3 domain comprises an α helix with a *C*-terminus tail (Figure 2). The α3 biorecognition helix is stabilized by helices 1 and 2 through hydrophobic contacts and forms a stabilized α-helix once DNA-bound. The helical conformation is essential for positioning the Arg^258^-Ile^262^-Val^269^ biorecognition triad to form a DNA-facing hydrophobic patch, critical for interactions with the methyl group of 5mC for ‘base readout’: Ile^262^ and Val^269^ appear to engage in hydrophobic methyl–methyl contact with the 5mC bases, stabilized by the hydrophobic contact between the aliphatic chain of Arg^258^ with Ile^262^ (Figure 2B). Notably, the helicity of α3 is further stabilized by a solvent-exposed Arg^267^/Glu^271^ salt bridge, while its *C*-terminus tail is not involved in DNA binding.

**Figure 2:**
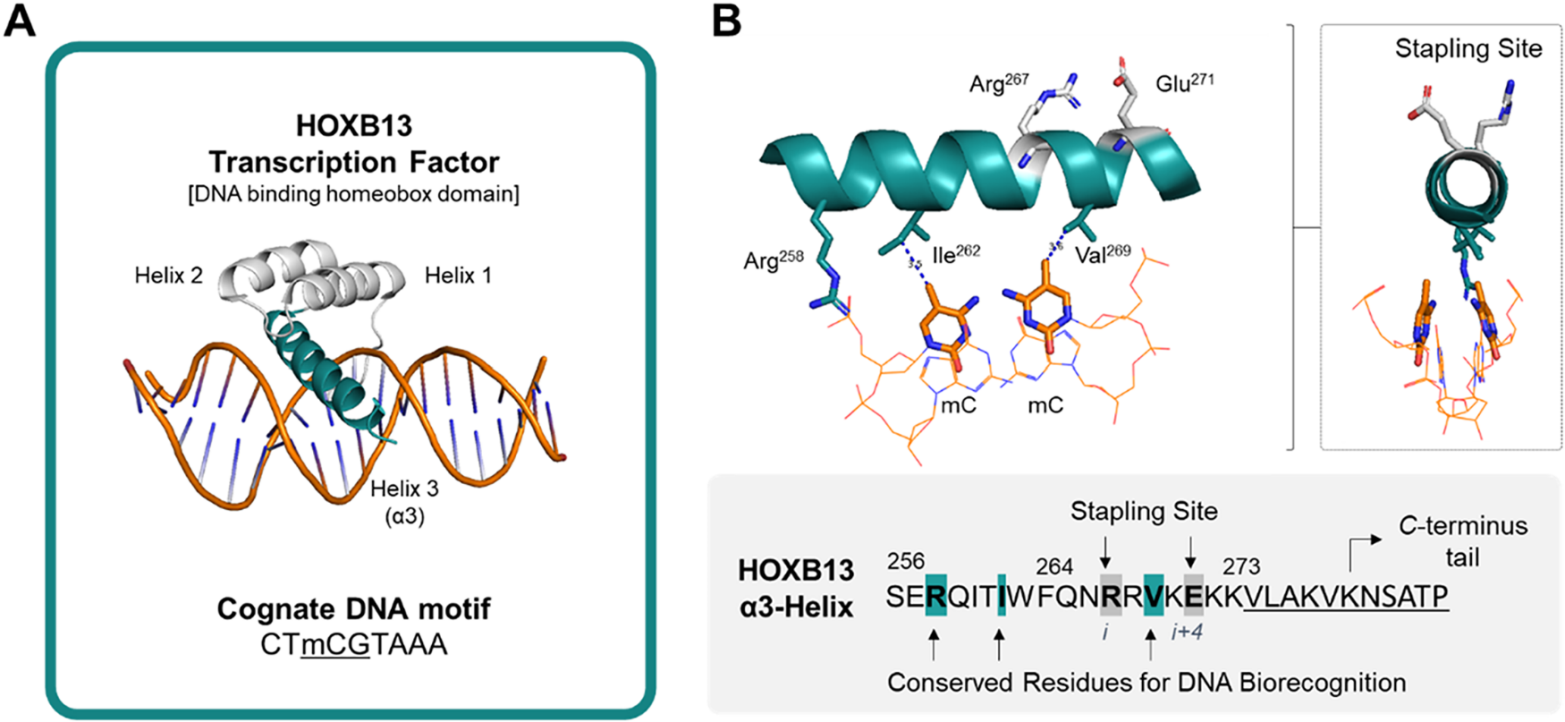
Key interactions of HOXB13 with its cognate methylated DNA motif. (A) Crystal structure of HOXB13 in complex with its methylated DNA-binding site. α3 recognition helix indicated in cyan (Protein Data Bank Entry: 5ef6). (**B)** Key interactions of DNA-facing α3 residues with 5-methylcytosine, and Arg-Glu salt bridge at the solvent-exposed face of the helix.

Using α3 as the blueprint, we created an 18-mer native peptide P1 (Ser^256^-Lys^273^) and stapled metallopeptide variants (MP2–MP10) (Figure 3). In all variants, the biorecognition triad was conserved and/or optimized for DNA binding and the *C*-terminus tail shortened. In the absence of tertiary stabilizations, unfolding of the α3 motif in aqueous solutions is expected due to competing hydrogen bonding interactions with water. To reduce entropy costs for helical folding required for DNA binding, the Arg^267^/Glu^271^ salt bridge was reconfigured with Asp^267^/Asp^271^ (MP2) to install a dirhodium metal staple for helicity stabilization. We further replaced selected residues to enhance helicity and methyl sensitivity. Solvent-exposed Ile^260^ (MP3), Glu^257^ (MP4), or Trp^263^ (MP5) were replaced by helicogenic α-aminoisobutyric acid^23^ (refer herein as Aib or X) to increase helical propensity. Val^269^ that recognizes the 5-methylcytosine on the CTmCGTAAA sequence was replaced by Leu (MP6), Thr (MP7), or Phe (MP8) to study the preference of apolar, polar, or aromatic residues for specific contact with 5mC over cytosine. Finally, C- and/or N-terminal truncation was also investigated (MP9-MP10) to determine the effective length for DNA biorecognition (Figure 3D).

**Figure 3:**
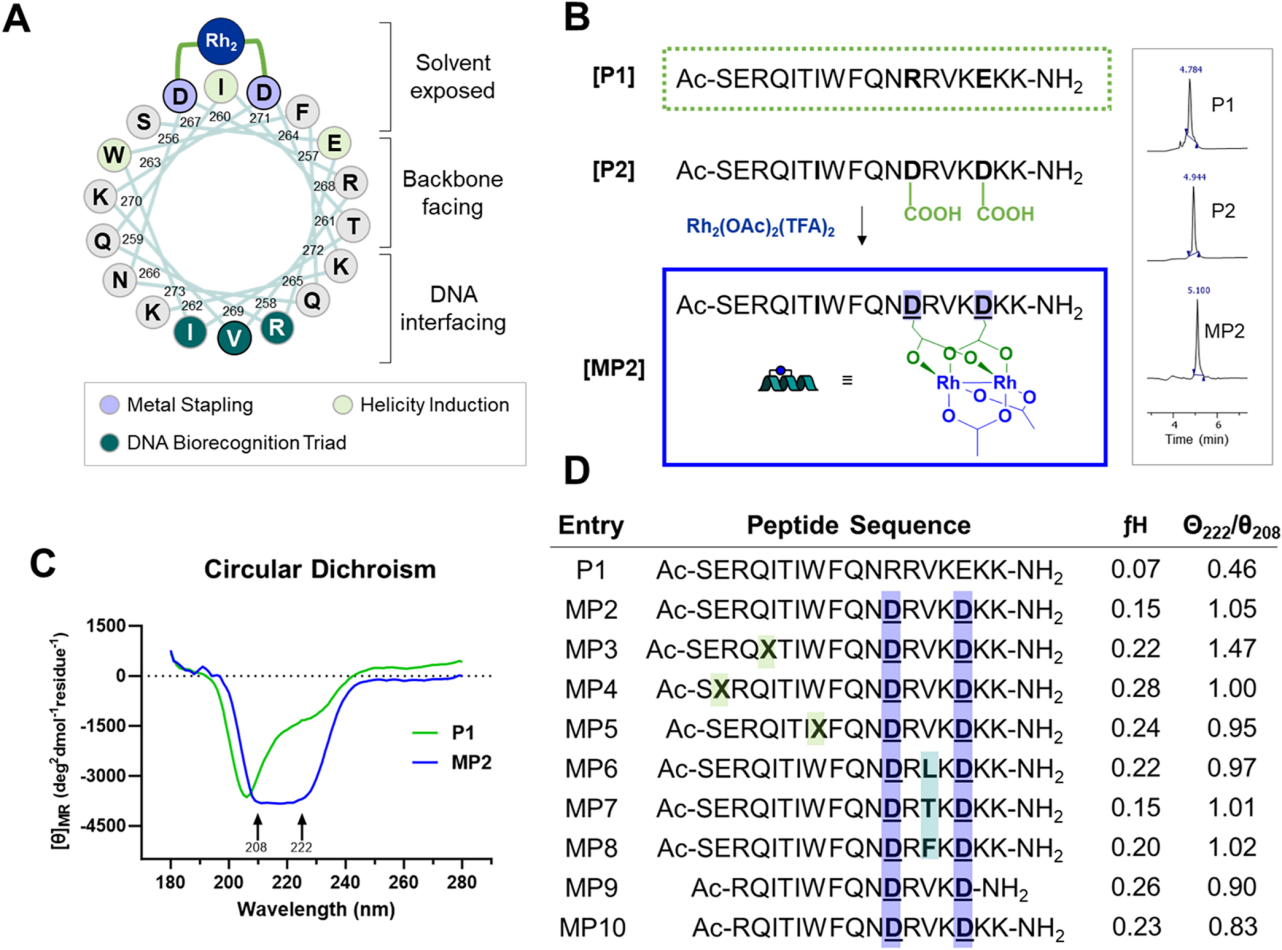
Design and assembly of stapled metallopeptides. (A) Helical wheel illustration of 18-mer (Ser^256^-Lys^273^) α3-derived stapled metallopeptide MP2. DNA biorecognition triad (teal), stapling site (lilac), and mutation sites (green) for helicity are highlighted. (B) Assembly of stapled metallopeptide MP2. Reconfiguration of Arg^267^-Glu^271^ (P1) to Asp^267^-Asp^271^ (P2) enabled chelation of dirhodium complexes to give metallopeptide (MP2). Stapled peptide MP2 (RT 5.10 min) displayed higher hydrophobicity than linear precursor P2 (RT 4.94 min) or P1 (RT 4.78 min), as seen in LC-MS analysis. (C) Circular dichroism spectra of unmodified P1 (random coil) and stapled MP2 (α helical) in 10 mM potassium phosphate solution (pH 7.4). (**D**) Helical content and α-helicity characterization of peptide library, where X = α-aminoisobutyric acid. Fractional helicity (fH) determined from the mean residue ellipticity at 222 and 222 nm/208 nm ratio.

### Assembly of HOXB13-Derived Metallopeptide Mimetics

The metallopeptides were synthesized based on a reported stepwise approach.^24^ Linear parent peptides (P1–P10) were synthesized using standard Fmoc solid-phase peptide synthesis (SPPS). A global deprotection and resin cleavage followed by solution-phase complexation with Rh_2_(OAc)_2_(TFA)_2_ successfully afforded the stapled metallopeptides (MP2–10) (see Section S2). Circular dichroism (CD) was used to determine peptide secondary structure and helicity content. The P1 control peptide adopted a random coil conformation in aqueous potassium phosphate buffer. CD spectra showed that at 25 °C and pH 7.4, native α3 in isolation (P1) displayed a low level of helical stability (fH = 7%) and a 222 nm/208 nm ratio of 0.46. In stark contrast, all metallopeptides displayed minima at 222 and 208 nm with improvement of helical content ranging from 15% to 28% and a 222 nm/208 nm ratio close to 1, indicative of α-helicity. Specifically, introduction of the dirhodium metal staple to MP2 led to a 2-fold improvement in helicity (15%). Adding an Aib helical promoter into MP3-MP5 (22–28%) further improved helicity, while the introduction of methyl-contact residues (MP6-MP8) or sequence truncation (MP9-MP10) did not affect helical stability.

### Metallopeptides Bind with Nanomolar Affinities to the Primary DNA-Binding Site of HOXB13

We proceeded to evaluate the binding affinity of P1 and the stapled metallopeptide variants for the target methylated DNA motif of HOXB13 (see Section S5.3). Building upon reported fluorescence quenching assays^25,26^ for DNA ligand binding, we elected to establish a fluorescent quenching assay to profile DNA-peptide interactions in our quantitative analysis. Balasubramanian and co-workers previously reported fluorescent dye quenching by guanine-rich oligonucleotides for direct measurement of proximal ligand binding to G-quadruplexes.^25^ This phenomenon can be attributed to photoinduced electron transfer between the interactions of guanine nucleobases and an excited fluorophore.^26,27^

Here, the binding affinity of the peptides was profiled against a AlexaFluor488^tm^-labeled methylated target DNA (MTG-AF488) bearing the primary biorecognition motif of HOXB13 (Figure 4A). MTG-AF488 is a fluorescent-labeled dsDNA composed of (1) an unlabeled oligonucleotide strand comprising an internal CTmCGTAAA consensus motif and guanine bases at its 5′-terminus; (2) a complementary oligonucleotide strand strategically labeled with AF488 at its 3′-position. Analogous to Balasubramanian’s approach,^25^ we envisioned that proximal peptide binding to the dsDNA can be measured via quenching of the AF488 fluorophore by adjacent guanines. The binding of basic peptides, such as MP2, can neutralize the DNA polyanion backbone and weaken the repulsive forces within the backbone of the dsDNA.^28^ This ligand-induced flexibility will enable the 5′-guanines to quench the complementary 3′-AF488 excited-state fluorophore in the vicinity.

**Figure 4:**
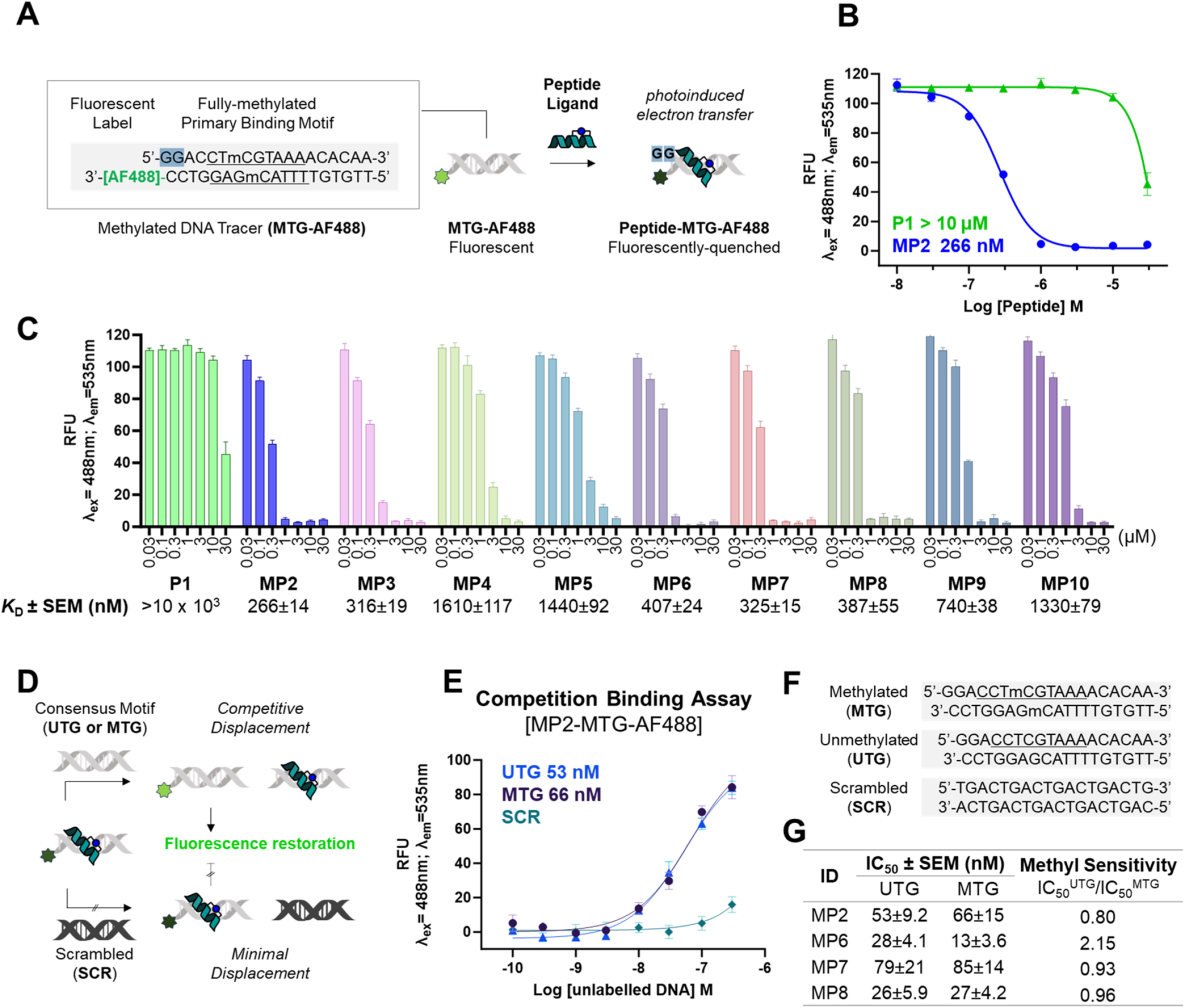
Binding affinity profiling of P1 and metallopeptides MP2-MP10. (A) The use of construct of MTG-AF488 as a fluorescent tracer. Proximal peptide binding was measured by guanine-mediated fluorescence quenching of AF488 fluorophore. (B) Binding affinities of peptides measured by fluorescence quenching assays between MTG-AF488 DNA and linear P1 or helical MP2. (C) Dose–response fluorescence quenching assays between MTG-AF488 DNA and all peptide variants (P1, MP2-MP10). (D) Competition assay measured by fluorescent restoration assays by titrating Peptide-MTG-AF488 complexes with unlabeled DNA competitors. (E) IC_50_ of unlabeled DNA competitors containing primary consensus (MTG or UTG) and nonconsensus (SCR) motif titrated against labeled MTG-AF488-MP2 complexes. (F) Sequence identity of UTG, MTG, and SCR unlabeled oligonucleotide competitors. (G) IC_50_ of unlabeled DNA competitors containing methylated (MTG) or unmethylated consensus (UTG) motifs titrated against labeled MTG-AF488 complexed with MP2, MP6, MP7, or MP8. All assays performed in at least three independent replicates (*n* = 3).

Indeed, MP2 bound strongly to MTG-AF488, leading to an increase in fluorescence quenching (Figure 4B) in a dose-dependent manner (*K*_D_ 266 nM). Conversely, minimal fluorescence quenching was observed for P1, indicative of a lack of binding affinity for MTG-AF488. As helices 1 and 2 of HOXB13 are critical for structural stabilization of α3, this suggests that the entropy cost of the destabilized P1 for helical folding was a key barrier for efficient binding to DNA. The binding affinities of all metallopeptide variants were further profiled (Figure 4C). While Aib replacement at Ile^260^ (MP3), Glu^257^ (MP4) or Trp^263^ (MP5) led to enhanced helical propensity, no improvement of binding affinity was observed. Aib incorporation at Glu^257^ or Trp^263^ position led to deleterious effects, while replacement at solvent-exposed Ile^260^ position was well-tolerated. Crystal structure analysis showed that while all three residues do not directly interact with the DNA bases, Trp^263^ and Glu^257^ are in proximity with the DNA backbones (see Section S7, Figure S5). It was postulated that Aib-induced rigidification of the peptide backbone may lead to unfavorable steric clashes with the DNA backbone at these sites.

The use of Leu, Thr, or Phe (MP6-MP8) in lieu of Val^269^ (MP2) for targeted contact with 5mC did not improve binding affinity. Notably, the incorporation of OH-containing Thr into the hydrophobic patch was well-tolerated. On the other hand, peptide truncations were detrimental, where significant loss of binding affinity was observed for MP9 and MP10. This suggests that the 18-residue helical core of α3 represents the minimal binding motif essential to maintain key interactions with the consensus binding motif. Overall, all stapled metallopeptides (*K*_D_ = 266 to 1610 nM) showed improved binding affinities compared to the unmodified P1 linear counterpart (*K*_D_ > 10 μM), albeit with lower affinities compared to the full-length HOXB13 (Δ*G* = −10.824 kcal/mol; *K*_D_ ≈ 8.5 nM).^7^

Hence, we have successfully downsized the HOXB13 protein to an 18-mer α3 peptide mimic that retains biorecognition ability to bind its cognate methylated DNA sequence. To the best of our knowledge, the above results represent the first examples of methylated DNA biorecognition by stapled peptides and should foster further research in the development of synthetic methylated DNA binders.

### Competition Assays Confirm MP2 Can Bind to DNA in a Sequence-Specific Manner

We proceeded to examine the sequence specificity of MP2 for DNA biorecognition using a competitive binding assay. Previous isothermal titration calorimetry (ITC) experiments by Taipale and co-workers indicated that HOXB13 protein can bind to its consensus motif in unmethylated and methylated states, with a preference for the latter.^7^ Here, unlabeled DNA motifs bearing a methylated (MTG) or unmethylated (UTG) binding site, as well as a nonconsensus scrambled (SCR) sequence, were used as competitive inhibitors to displace MTG-AF488 from MP2. 10 nM MTG-AF488 and 550 nM MP2 (which gave 60% of the maximum response based on K_D_ 266 nM) were selected for use in the competition assay.

Titrating in UTG or MTG led to fluorescence restoration in a dose-dependent manner, indicating competitive displacement of MTG-AF488 from MP2, whereas scrambled SCR did not fully annul fluorescence quenching, with only <20% fluorescence restored even at the highest titration value (Figure 4E). The weak association between MP2 and nonconsensus motif SCR is indicative of nonspecific electrostatic association, a well-established phenomenon where TFs can interact nonspecifically with DNA while scanning for their consensus motifs.^29,30^ Using direct fluorescence quenching as an indicator of proximal ligand binding, these competition results are indicative of sequence-specific and reversible binding of MP2 to the labeled methylated DNA motif. The simplified assay platform enabled rapid profiling of methyl sensitivity. The IC_50_ of unmethylated UTG and methylated MTG for the inhibition of MTF-AF488 binding to MP2 were determined as 53 nM and 66 nM, respectively. These observations indicate that MP2 can achieve HOXB13-like binding to both methylated and unmethylated consensus DNA motifs. However, MP2 did not discriminate between UTG and MTG, in contrast to the previous report by Taipale and co-workers where the native HOXB13 protein preferentially binds the methylated binding site over the unmethylated site with ∼3.9-fold preference.^7^ Reported crystal structures of the native HOXB13-DNA complexes suggest that the methyl group of Val^269^ can form favorable methyl–methyl contact with 5mC and weaker contact with cytosine (Figure 5B). While we have demonstrated that miniaturization of HOXB13 to a constrained α3 mimic can retain native-like biorecognition for its consensus motif, helices 1 and 2 may be required for additional contacts for base-readout and/or optimal structural stabilization of α3 for specific methylated DNA shape-readout.

**Figure 5:**
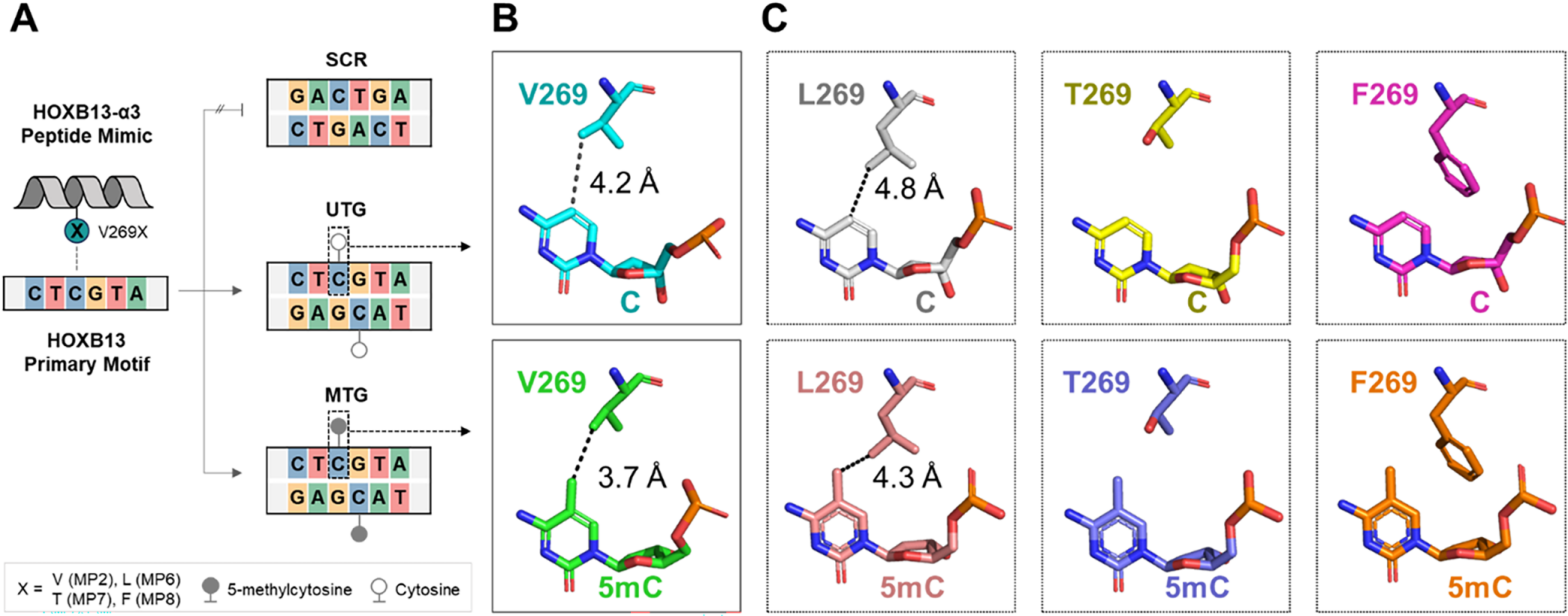
Comparison of key interactions between V269X residue and 5mC for methyl sensitivity. (A) Schematic illustration of key interactions between V269X (X = V, L, T or F) of α3 mimic with the primary DNA-binding motif of HOXB13. (B) Interactions of Val269-HOXB13 with unmethylated binding motif (top; Protein Data Bank Entry: 5edn) or methylated primary binding motif (bottom; Protein Data Bank Entry: 5ef6). (C) Predicted interactions of mutated HOXB13-DNA complexes using structural models generated by AlphaFold3. V269X variants (X = Leu, Thr, or Phe) of HOXB13 complexed with unmethylated (top row) and methylated cytosine (bottom row).

### V269X Peptide Mutants Can Enhance Methyl Sensitivity through Hydrophobic Contact with 5-Methylcytosine

We further compared the methyl sensitivity of MP2 (V269) against other mutants: MP6 (V269L), MP7 (V269T), and MP8 (V269F). Methyl sensitivity of the V269X mutants was examined by comparing the IC_50_ of unlabeled UTG or MTG competitors for the displacement of their complexes with MTG-AF488. Of all the mutants, only the V269L mutant exhibited a modest preference for methylated binding site (IC_50_^UTG^/IC_50_^MTG^ = 2.15) (Figure 4G).

Alphafold3-predicted^31^ structural models of HOXB13-V269X mutants complexed with methylated or unmethylated primary DNA-binding motif were generated to elucidate their associated mechanism for methyl sensitivity (Figure 5C). Notably, the orientation of the elongated Leu^269^ side chain of MP6 was projected slightly further due to steric effects (Figure 5C). The methyl–methyl contact between the methyl group of Leu^269^ and 5mC (4.3Å) can be maintained as the pair lies within the typical distance of CH···CH interactions (3.7–4.4Å).^32^ Conversely, potential CH/π interaction of Leu and cytosine cannot be attained as they were positioned beyond the typical distance of π···CH interaction (3.8–4.4 Å). In addition, MP6 (fH = 22%) exhibited enhanced helicity stabilization compared to MP2 (fH = 15%). This may explain the improved binding preference of Leu-bearing MP6 for the methylated binding site observed herein. Importantly, MP6 exhibited a preference for methylated binding site (2.15-fold) within the range of a methyl-specific MBD protein, MeCP2 (2.7-fold).^33^

On the other hand, MP7 and MP8 mutants, where Val^269^ was replaced by Thr or Phe, respectively, displayed no preference for 5mC over cytosine (Figure 5C). Previous *in silico* analysis^34^ suggested that favorable recognition of 5mC can be targeted using polar residues (e.g., Thr, Ser) through H-bonding interactions with the N4 and O2 groups of 5mC, and aromatic (e.g., Phe, Tyr) amino acids through π-based interactions. Here, the introduction of OH-containing Thr or aryl Phe was found to maintain similar methyl sensitivity as Val^269^, with no notable improvement in 5mC preference. No H-bonding interactions between Thr and 5mC were noted, reminiscent of the previously reported cocrystallized structure of the DLX3 homeodomain protein with methylated DNA.^7^ DLX3 bearing a OH-containing Ser at the same position was shown to display a weak preference for 5mC due to decreased hydrophobicity. For the V269F mutant, nonspecific edge-to-face pi-stacking interactions between the Phe aromatic ring and both 5mC and cytosine were noted. Previous molecular dynamics simulation studies revealed that the stability of the water network at the DNA–protein interfaces is a key contributor to differential HOXB13 binding activity.^21^ We cautiously note that prediction models cannot reproduce the dynamic behavior of biomolecules in solution; therefore, future experimental validations are needed to resolve the local DNA base-amino acid contacts conclusively.

### MP2 Metallopeptide Mimetic Is Proteolytically Stable

The proteolytic stability of the metallopeptide MP2 was compared to that of unmodified peptide P1 using a trypsin digestion assay (Section S9). Degradation of P1 by trypsin occurs almost immediately, with the formation of a new peptide fragment peak after 5 min of incubation. Multiple cleavage sites containing a preferred Arg residue at the P1 position were recognized by trypsin: Arg^268^-Val^269^, Arg^258^-Gln^259^, and Arg^267^-Arg^268^. Surprisingly, unexpected trypsin cleavage at the C-terminal of apolar Phe^264^ was also observed (Figure S7). On the other hand, minimal cleavage of MP2 was observed up to 2 h. We demonstrated that unmodified P1 linear peptide was more susceptible to proteolysis as proteases universally recognize extended β-stranded conformations,^35^ whereas helical metallopeptide MP2 poses superior resistance to protease hydrolysis. This finding highlights the potential use of the stapled peptides in future biological applications.

## Discussion

There has been a long-standing interest in delineating the interactions of methylated DNA motifs to study important epigenetic marks for diseases. Here we generate mimetic tools to dissect the recognition basis of methyl-plus HOXB13 protein for a methylated DNA biomarker, an interaction implicated in prostate cancer initiation. We first show that stapled metallopeptides are HOXB13-α3 mimetics that can recognize the cognate primary consensus DNA motif of native HOXB13 protein. We further validate that the mimetics can bind to both unmethylated and methylated consensus motifs, in agreement with a previous report.^7^ We then examine the methyl sensitivity of V269X mimetic mutants for preferential 5-methylcytosine recognition through hydrophobic contacts. Previous analysis^36^ by Rohs and co-workers suggested that methylated and unmethylated DNA adopt similar overall geometries during TF recognition, where hydrophobic contact with 5mC can merely fine-tune the binding specificities of TFs. Indeed, crystal structures of HOXB13 complexes revealed similar binding geometries with their methylated and unmethylated DNA cognate motifs (Section S7, Figure S6). Our studies further show that the V269L mutation can enhance methyl sensitivity through methyl–methyl contact with 5mC, with only a 2-fold improvement of methyl-specificity compared to native Val.

To date, the amino acid recognition basis for epigenetic alphabet 5mC remains inconclusive. 5mC can be recognized in different ways by amino acids.^37^ *In silico* analysis^34^ suggests recognition of 5mC can be favored by aromatic, polar, or basic side chains; however, limited experimental profiling of amino acid propensities for 5mC recognition exists. By isolating the individual contribution of the α3 DNA-binding domain to DNA biorecognition, our screening studies indicate that targeting a single CpG methylation site can productively enhance methyl sensitivity, and optimizing hydrophobic contacts with methyl groups can subtly enhance the methyl-specificity of TF. The quest for sequence- *and* methyl-specific DNA binders presents an open challenge. From our perspective, future work may involve the exploitation of unnatural methyl-contact amino acids to further enhance specific interactions with 5mC. It is likely that targeting adjacent 5mC sites for additional base-readout and accommodating the widened major groove of methylated DNA for specific shape readout will be required to provide new routes to create highly methyl-specific DNA binders.

## Conclusion

In summary, we have generated first-in-class methyl-sensitive mimetics to provide new insights into the local interactions of HOXB13 with its cognate methylated DNA motif, which is a cancer biomarker. The mimetics will serve as useful chemical tools to probe HOXB13-associated binding sites. Furthermore, the peptide reagents provide the opportunity to develop peptide microarrays as a simpler, complementary alternative to current protein microarrays for the profiling of TF-DNA interactions. Importantly, our miniaturization strategy will enable the creation of synthetically accessible dirhodium-stapled mimetics that are highly programmable for other methyl-plus transcription factors to study other DNA targets. We envision that these mimetics can potentially serve as useful tools to disrupt protein-methylated DNA interactions. This offers new opportunities to target and modulate risk gene interactions and a new starting point to inhibit oncogenic transcription associated with DNA methylation. Supported by enhanced proteolytic stability, the mimetics offer new opportunities for the investigation of transcriptional activities in living settings. Therefore, our methyl-sensitive, sequence-specific metallopeptides represent a new platform technology that will create new tools for epigenetic research that can help answer important questions in biology.

## Supplementary Material



## Notes

Experimental procedures for peptide synthesis; protocol and data for circular dichroism and fluorescence assays; compound characterization data (LC-MS, NMR); and proteolytic stability studies (PDF)
