# Supplementary material for: Miniaturized HOXB13 Mimetics Are Sequence-Specific, Methyl-Sensitive DNA Binders
Source: J Am Chem Soc. 2026 Jul 14;148(29):30785–92. doi: 10.1021/jacs.6c01290 (PMC13426251; doi:10.1021/jacs.6c01290)
Supplement: Supplementary file 1 [file ja6c01290_si_001.pdf]

Supporting information

Miniaturized HOXB13 Mimetics Are Sequence-Specific, Methyl-Sensitive DNA Binders

Claire Wong and Shiao Y. Chow*

Department of Pure and Applied Chemistry, University of Strathclyde

295 Cathedral Street, Glasgow G1 1XL, Scotland, U.K.

*E-mail: shiao.chow@strath.ac.uk

Table of Contents

S1. General information 2

S2. Synthetic procedures 3

S2.1 General Procedure 1: Linear Peptide Synthesis 3

S2.2 General Procedure 2: Assembly of metallopeptide 4

S3. Structure of Linear and Staple Peptides 6

S4. Circular Dichroism 7

S4.1 General Procedure for Circular Dichroism measurement 7

S4.2 Circular Dichroism Spectra 8

S5. Fluorescence Assay Profiling 9

S5.1 Library of dsDNA oligomers 9

S5.2 Assay Protocol 9

S5.3 Assay Data 10

S6. Characterisation Data of Peptides and Stapled Metallopeptides 12

S7. Key interactions of HOXB13 with primary DNA binding motif 15

S8. ^{1}H NMR and HPLC Data 16

S9. Proteolytic stability assay 27

S10. References 28

S1. General information

All raw materials used for peptide chain assembly were commercially sourced and used without further purification. Peptide grade solvents (dimethylformamide (DMF) and dichloromethane (DCM)), pyrrolidine and N-methyl-2-pyrrolidone (NMP) were sourced from Rathburn (Scotland, UK). All Fmoc protected L-amino acids, *N*,*N*,*N*′,*N*′-tetramethyl-*O*-(1*H*-benzotriazol-1-yl)uronium hexafluorophosphate (HBTU), triisopropylsilane (TIPS), trifluoroacetic acid (TFA), acetic acid, ethyl cyano(hydroxyimino)acetate (oxyma) and *N*,*N*′-diisopropylcarbodiimide (DIC) were sourced from Fluorochem. Rink MBHA low loading resin (100-200 mesh, 0.37 mmol/g) and diisopropylethylamine (DIPEA) was sourced from Sigma Aldrich. Peptides chain assembly was carried out using Fmoc synthesis strategies throughout on either a Supelco SPE Visaprep 12 vacuum manifold with 20 ml peptide reactor with PE frits (pore size 25 μm) sourced from Carl Roth or using CEM Liberty Prime 2.0 Automated Microwave Peptide Synthesis. Crude peptides were purified by reverse-phase HPLC using a Gilson preparative HPLC system of 322 pumps coupled to a 151 UV/Vis spectrometer, 234 Autoinjector and a GX-271 liquid handler using a Waters XBridge Prep OBD C18 19 x 50 mm, 5 μm column at room temperature. Purifications were performed using gradient methods ranging from 5–90% MeCN + 0.1% TFA and H_2O + 0.1% TFA over 25 minutes at a flow rate of 8 mL/min, with UV monitoring at 254 nm. DNA oligomers were commercially supplied from Integrated DNA Technologies (United States).

Commercially-available raw materials and solvents used for organic synthesis were used without further purification. Rhodium (II) acetate dimer (99.9% trace metal) was commercially supplied from Sigma Aldrich; solvents used were synthesis grade and sourced from Alfa Aesar, Sigma Aldrich and Acros Organics. Organic reactions were carried out under nitrogen atmosphere as standard and on a thermometer-controlled heating mantle. Thin layer chromatography (TLC) was conducted using pre-coated aluminium TLC plates, Polygram, SIL G/UV. Results were analysed under a UV lamp at λ=254 or 366 nm and stained using $KMnO_4$ where necessary. Purification of compounds was carried out using column chromatography where necessary using ZEOprep 60 HYD 40-63 μm silica gel.

All precursors and purified peptides were analysed by NMR, LCMS, and HRMS using the following standard methods.

NMR. NMR spectra were recorded on a Bruker Ultrashield 400 MHz or 500 MHz spectrometer at 25°C. Chemical shifts were recorded in parts per million (ppm) downfield from tetramethylsilane (TMS) and coupling constants in Hertz (Hz). ^{1}H NMR was referenced to residual solvent standard ($CDCl_3$ (7.26 ppm) or 90:10 H_2O:D_2O (4.71 ppm)). Water suppression experiments were used for analysis of peptides where O_1P values ~4.71 ppm. NMR data was processed using Mestrenova software and is represented by chemical shift, integration, multiplicity, coupling constant (Hz). The following abbreviations are used for multiplicities: s = singlet; d = doublet; t = triplet; q = quartet; quint. = quintet; dd = doublet of doublets; dt = doublet of triplets; m = multiplet; br = broad.

LC-MS. Liquid chromatography–mass spectrometry (LC-MS) was employed for monitoring reaction progress and assessing final product purity. Analyses were performed on an Agilent Technologies 1220 LC system coupled to an Agilent 6100 series quadrupole mass spectrometer operating in ESI/APCI mode. Chromatographic separation used a Poroshell 120 C18 column (4.6 × 75 mm, 2.7 μm) maintained at 40 °C. UV data was collected at 214 nm for peptide-containing samples and 254 nm for all other analytes. All compounds were analysed using an 18-minute method. Mobile phase: H_2O + 0.1% formic acid and MeCN + 0.1% formic acid. The gradient progressed from 5% to 100%

MeCN (+ 0.1% formic acid) between 1.4→ 8 mins, held at 100% MeCN (+ 0.1% formic acid) from 8 → 13.5 mins; returned to 5% MeCN (+ 0.1% formic acid) from 13.5 → 16.5 mins, then held at 5% MeCN (+ 0.1% formic acid) between 16.5 → 18 mins.

HRMS. HRMS was carried out on a UHPLC Thermo Scientific LTQ orbitrap (Thermo scientific, Waltham, MA) equipped with a Kinetex 2.6 µm C18 100 Å LC column (50 x 2.1 mm) under conditions of positive electrospray ionisation. Analysis was conducted using a 4-minute method with a mobile phase of MeCN (+ 0.1% formic acid) and H_2O (+ 0.1% formic acid).

S2. Synthetic procedures

S2.1 General Procedure 1: Linear Peptide Synthesis

Synthesis of linear peptides

Peptides were synthesized using standard solid-phase synthesis protocols via Fmoc chemistry on Rink MBHA low loading resin (0.37 mmol/g) using manual or automated peptide synthesis:

Manual Peptide Synthesis. Peptides were synthesised using a Supelco SPE Visaprep 12 vacuum manifold and 20 ml peptide reactor with PE frits (pore size 25 µm) sourced from Carl Roth. All coupling and deprotection was carried out at room temperature. Rink MBHA low loading resin (0.05 mmol scale) was swelled in DMF (2 ml) for 1 h then deprotected with 1 ml 20 % piperidine in DMF for 20 mins. Fmoc L- amino acids (4 equiv.) were preactivated in a separate vial using HBTU (4 equiv.) and DIPEA (6 equiv.) in DMF (0.5 M reaction concentration) for 5 mins before addition to the resin. Fmoc groups were deprotected using 20% piperidine in DMF for 20 mins, coupling and deprotection processes were repeated until peptide was grown to desired length. Following the final coupling and deprotection, N-termini of linear peptides were acetylated using a preactivated solution of acetic acid (5 equiv.), HBTU (5 equiv.) and DIPEA (5 equiv.) in DMF (0.5 M reaction concentration) for 1 h. Coupling and deprotection were monitored using Kaiser Test qualitative method and the resin was washed with copious amounts of DMF and DCM following each coupling, deprotection and acetylation.

Automated Peptide Synthesis. Peptides were synthesised using CEM Liberty Prime 2.0 automated Microwave Peptide Synthesis. Rink MBHA low loading resin (0.1 mmol scale) was swelled in DMF then Fmoc deprotected using 25% pyrrolidine in DMF at 105 °C for 5 mins. Fmoc L- amino acids were prepared in 15% NMP in DMF and coupled using DIC (0.75 M) and oxyma (0.26 M) at 110 °C for 40 seconds. Fmoc-amino groups were deprotected using 25% pyrrolidine in DMF at 105 °C for 5 mins. The coupling and deprotection processes were repeated until peptide was grown to desired length. Following the final coupling and deprotection, N-termini of linear peptides were acetylated using a preactivated solution of acetic acid (5 equiv.), HBTU (5 equiv.) and DIPEA (5 equiv.) in DMF (0.5 M reaction concentration) for 1 h and monitored using Kaiser Test qualitative method. The resin was washed with DMF following each coupling, deprotection and acetylation.

Peptide cleavage and Purification

The peptide was subjected to global cleavage from the resin using a cocktail of TIPS:H_2O:TFA (0.5:0.5:9) (10 ml per gram of resin) for 3 hours. The mixture was filtered into cold diethyl ether (50 ml) and the resulting suspension was centrifuged to obtain a pellet. The resulting precipitation was washed twice with cold diethyl ether and supernatant discarded each time to give the crude peptide as a solid. The crude mixture was redissolved in 50:50 MeCN:H_2O (with a maximum of 10% TFA for

insoluble peptides) and subsequently purified by a Gilson preparative HPLC system using a Waters XBridge Prep OBD C18 19 x 50 mm, 5 µm column at room temperature. A gradient from 5–90% MeCN + 0.1% TFA and H_2O + 0.1% TFA over 25 minutes at a flow rate of 8 mL/min, with UV monitoring at 254 nm was used. Lyophilization of relevant fractions gave desired product as a white solid.

S2.2 General Procedure 2: Assembly of metallopeptide

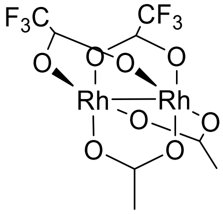

(1)

Preparation of Dirhodium Precursor *cis*-$Rh_2(OAc)_2(TFA)_2$ (1)

$Rh_2(OAc)_2(TFA)_2$ was synthesised according to reported methods.[1] To an oven dried vial, kept under nitrogen atmosphere was added $Rh_2(OAc)_4$ (15.2 mg, 0.034 mmol, 1 equiv.). The vial was purged with nitrogen for 30 mins before TFA (2 mL) was added. The resulting teal-blue solution was stirred at room temperature for 2h. After 2h, the mixture was concentrated under reduced pressure to remove excess TFA and purified by normal phase chromatography (toluene/MeCN = 9:1 to 4:1) to give a blue solid as the acetonitrile adduct product. Yield: 41% (8 mg). TLC R_f = 0.26 (9:1 toluene:MeCN). ^{1}H NMR (400 MHz, $CDCl_3$): δ 2.56 (3H, s, coordinated MeCN), 2.00 (6 H, s, OAc-CH_3). ^{19}F NMR (400 MHz, $CDCl_3$): δ -74.54 (s). MS (ESI+): calculated for $C_8H_6F_6O_8Rh_2$ $[M+H]^+$ 550, found: 632.90 $[M+2MeCN+H]^+$.

Spectral data are in agreement with previous report.[1]

Preparation of Metallopeptides MP2-MP11

$Rh_2(OAc)_2(TFA)_2$ was complexed with parent peptides P2-P10 using a modified literature procedure to obtain corresponding metallopeptides.[2] Linear peptides **P2-P10** (4 to 7 µmol, 1 equiv.), $Rh_2(TFA)_2(OAc)_2$ (1 equiv.) and 128 mM 2-(*N*-Morpholino)methanesulfonic acid (MES) (3 mM reaction concentration) buffer was stirred at 50 °C for 3h and monitored for the disappearance of $Rh_2(TFA)_2(OAc)_2$ by LCMS. Following completion, the reaction mixture was purified by direct injection of the reaction mixture onto reverse-phase Gilson HPLC using a gradient of 5–90% MeCN + 0.1% TFA and H_2O + 0.1% TFA over 25 minutes. Relevant fractions were lyophilised to give the final product as a pale blue solid. Final compounds were analysed by LCMS, HRMS and NMR.

Confirmation of complexation via ^{1}H NMR analysis

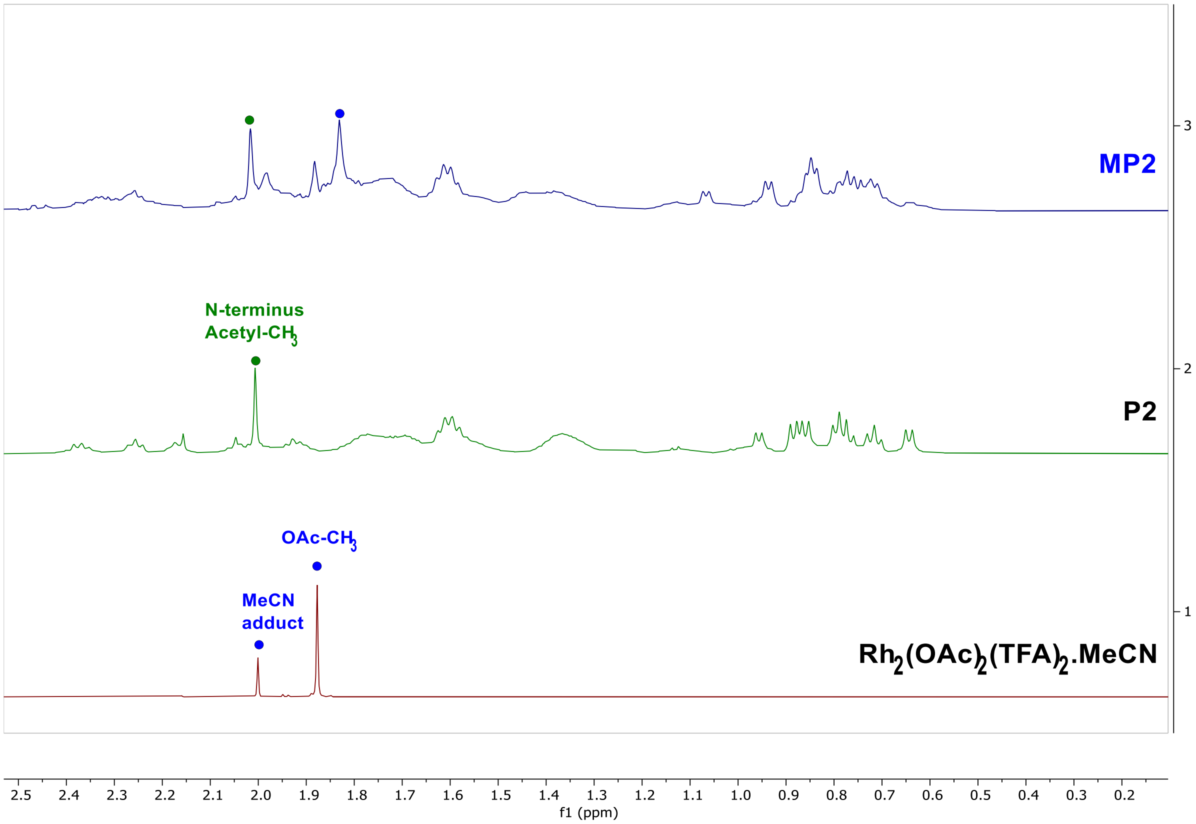

Figure S1. ^{1}H-NMR in 90:10 H_2O/D_2O of metallopeptide MP2 (top), parent peptide P2 (middle) and dirhodium complex precursor, $Rh_2(OAc)_2(TFA)_2$.MeCN (bottom).

S3. Structure of Linear and Staple Peptides

Ac-SERQITIWFQNRRVKEKK-NH_2 (**P1**)

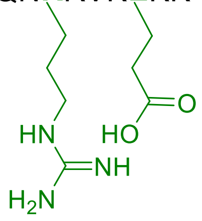

Ac-SERQITIWFQNDRVKDKK-NH_2 (**MP2**)

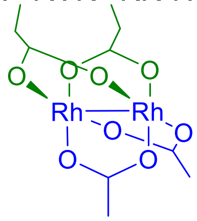

Ac-SERQXTIWFQNDRVKDKK-NH_2 (**MP3**)

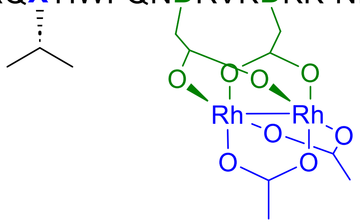

Ac-SXRQITIWFQNDRVKDKK-NH_2 (**MP4**)

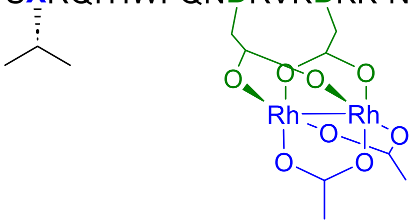

Ac-SERQITIXFQNDRVKDKK-NH_2 (**MP5**)

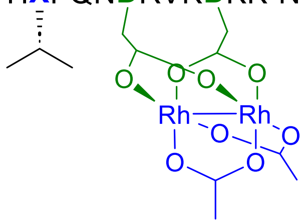

Ac-SERQITIWFQNDRLKDKK-NH_2 (**MP6**)

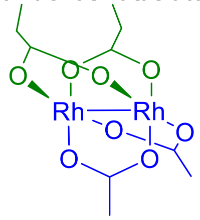

Ac-SERQITIWFQNDRTKDKK-NH_2 (**MP7**)

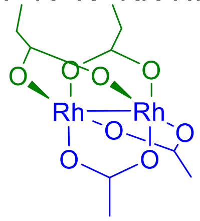

Ac-SERQITIWFQNDRFKDKK-NH_2 (**MP8**)

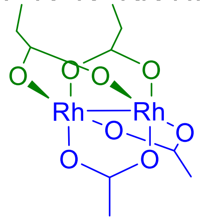

Ac-RQITIWFQNDRVKD-NH_2 (**MP9**)

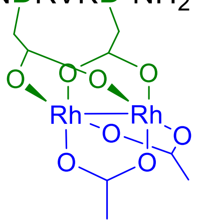

Ac-RQITIWFQNDRVKDKK-NH_2 (**MP10**)

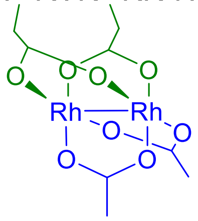

S4. Circular Dichroism

S4.1 General Procedure for Circular Dichroism measurement

50 μM peptide samples were prepared in 10 mM potassium phosphate solution (pH 7.4). Accurate concentration of peptide samples was first measured using PULCON NMR method.[3] CD measurements were performed in a 2 mm quartz cell (Hellma Analytics) at 25°C; wavelength range 185 – 260 nm; bandwidth of 1.0nm; response time of 1s; resolution step width of 1 nm and in triplicate using a Chirascan Circular Dichroism spectrometer and processed using Software 2.0.2 (Applied Photophysics Ltd ANM System V2.0.1). Baseline subtraction and smoothing were performed using the in-built software. Raw data was converted from millidegrees to mean residual ellipticity using built-in software from path length (2 mm), concentration (50 μM) and number of amino acids in the peptide. Spectra were analysed using Graphpad Prism Software.

Fraction helicity (f_H) was calculated based on mean residual ellipticity at 222 nm. This follows the assumption by Baldwin and Luo that alpha helical content is linearly related to ellipticity at 222 nm.[4] This calculation relies on determining the random coil $[\theta]_c$ and infinite alpha-helix $[\theta]_{\infty 222}$.

$$f_H = \frac{[\theta]_{222} - [\theta]_c}{[\theta]_{\infty 222} - [\theta]_c}$$

$$[\theta]_c = 2220 - 53T$$

$$[\theta]_{\infty 222} = (-44\,000 + 250T)\left(1 - \frac{x}{N_p}\right)$$

Where:

$[\theta]_{222}$ = mean residual ellipticity at 222 nm in deg cm^2 $dmol^{-1}residue^{-1}$.
$[\theta]_c$ is the mean residual ellipticity of the peptide in a random coil conformation.[5]
$[\theta]_{\infty 222}$ is the maximum theoretical mean residue ellipticity for a helix of n residues.[5]
T = temperature in degrees Celsius
N_p = number of peptide units
x = number of non-H-bonded peptide CO groups in a carboxyamidated peptide described by Luo and Baldwin[4] (we used x=3).

S4.2 Circular Dichroism Spectra

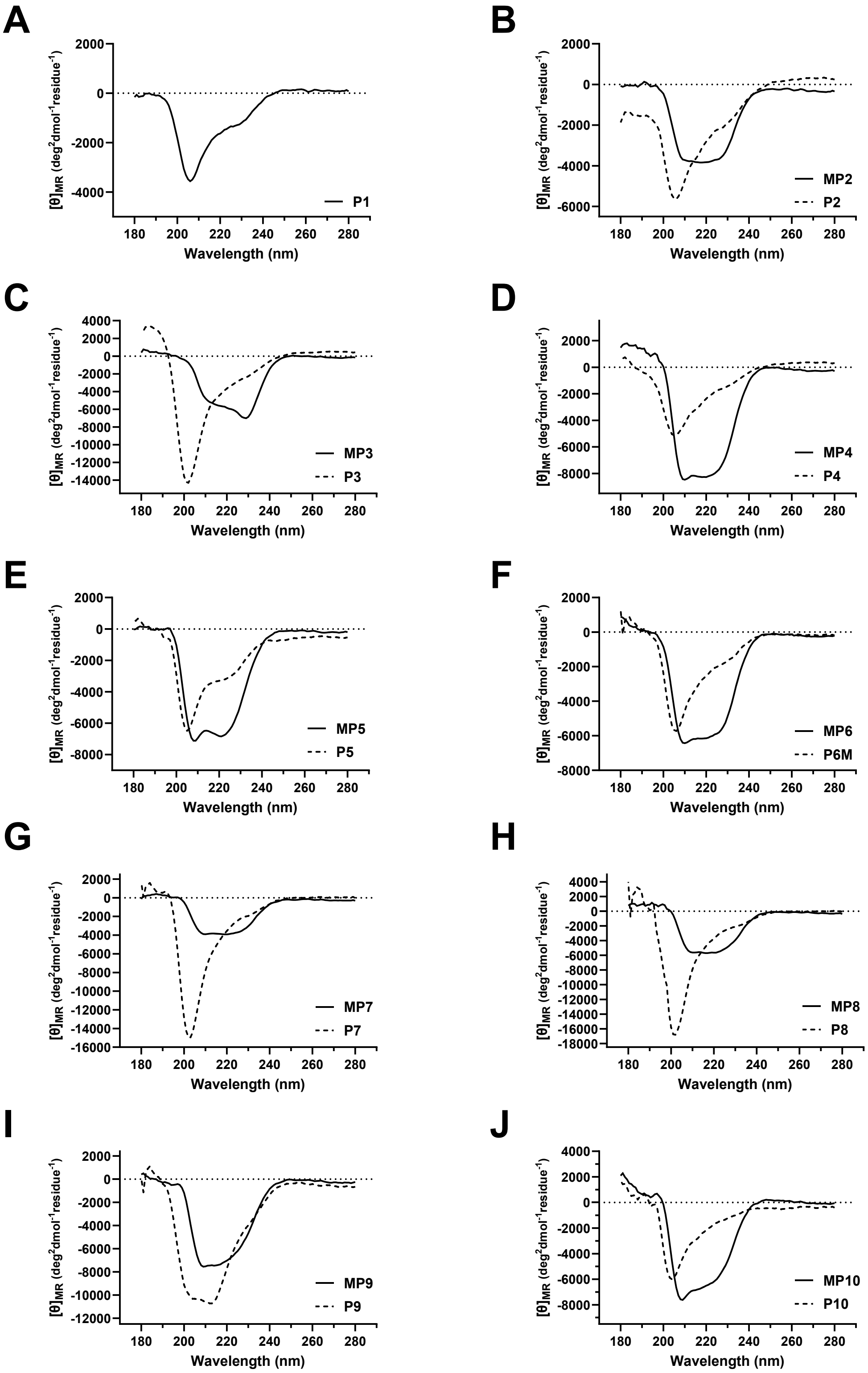

Figure S2. CD spectra of peptide library. (**A**) P1; (**B**)-(**J**) stapled metallopeptides (solid line; MP2-MP10) and their corresponding linear parent peptides (dotted line; P2-P10).

S5. Fluorescence Assay Profiling

S5.1 Library of dsDNA oligomers

Oligonucleotide duplexes were custom ordered from Integrated DNA Technologies (USA). Alexafluor488™ fluorophore was appended onto the 3'-end of the complementary strand of the target DNA sequence to create an 18-mer double-stranded methylated DNA fluorescence tracer (MTG-AF488). Unlabeled double-stranded DNA competitors containing methylated (MTG) or unmethylated (UTG) consensus motif, as well as non-consensus scrambled motif (SCR) were used in competition assays. The identity of oligonucleotide duplexes used in binding affinity and competition assays were as follows:

5'-GGACCTmCGTAAAACACAA-3'<br>3'-**[AF488]**-CCTGGAGmCATTTTGTGTT-5'	Labelled Methylated Target Motif<br>(**MTG-AF488**)
5'-GGACCTmCGTAAAACACAA-3'<br>3'-CCTGGAGmCATTTTGTGTT-5'	Methylated Target Motif<br>(**MTG**)
5'-GGACCTCGTAAAACACAA-3'<br>3'-CCTGGAGCATTTTGTGTT-5'	Unmethylated Target Motif<br>(**UTG**)
5'-TGACTGACTGACTGACTG-3'<br>3'-ACTGACTGACTGACTGAC-5'	Scrambled Control<br>(**SCR**)

S5.2 Assay Protocol

Binding affinity assay

The binding affinity (K_D) for peptide-DNA interactions was determined using fluorescent quenching assay. Alexafluor488-labelled methylated target motif (**MTG-AF488**) (10 nM) was titrated with peptide of interest (30 µM to 10 nM) in 10 mM Tris/HCl pH 7.5, 50 mM NaCl, 2 mM $MgCl_2$, 0.01% Tween-20 (v/v) at 25°C. The assay was performed in a 96 well Black Optiplate plate with a clear bottom. After 2 h incubation, fluorescence intensity (λ_{ex} = 488 nm; λ_{em} = 535 nm) was measured using CLARIOstar *Plus* multimode microplate reader from BMG LabTech. Data was processed using GraphPad Prism 10 to determine K_D values. Assays were performed in three independent triplicates.

Competition assay

MTG-AF488 (10 nM) and metallopeptide (550 nM) were pre-incubated in 10 mM Tris/HCl pH 7.5, 50 mM NaCl, 2 mM $MgCl_2$, 0.01% Tween-20 (v/v) in a 96 well Black Optiplate plate with a clear bottom at 25°C. The MTG-AF488-metallopeptide complexes were titrated with unlabelled DNA competitors UTG, MTG or SCR (300 nM to 100 pM); displacement of MTG-AF488 was quantified by fluorescence restoration. Fluorescence intensity (λ_{ex} = 488 nm; λ_{em} = 535 nm) was measured using CLARIOstar *Plus* multimode microplate reader from BMG LabTech. Data was processed using GraphPad Prism 10 to determine IC_{50} values. Assays were performed in three independent triplicates.

S5.3 Assay Data

S5.3.1 Fluorescent Quenching Assay

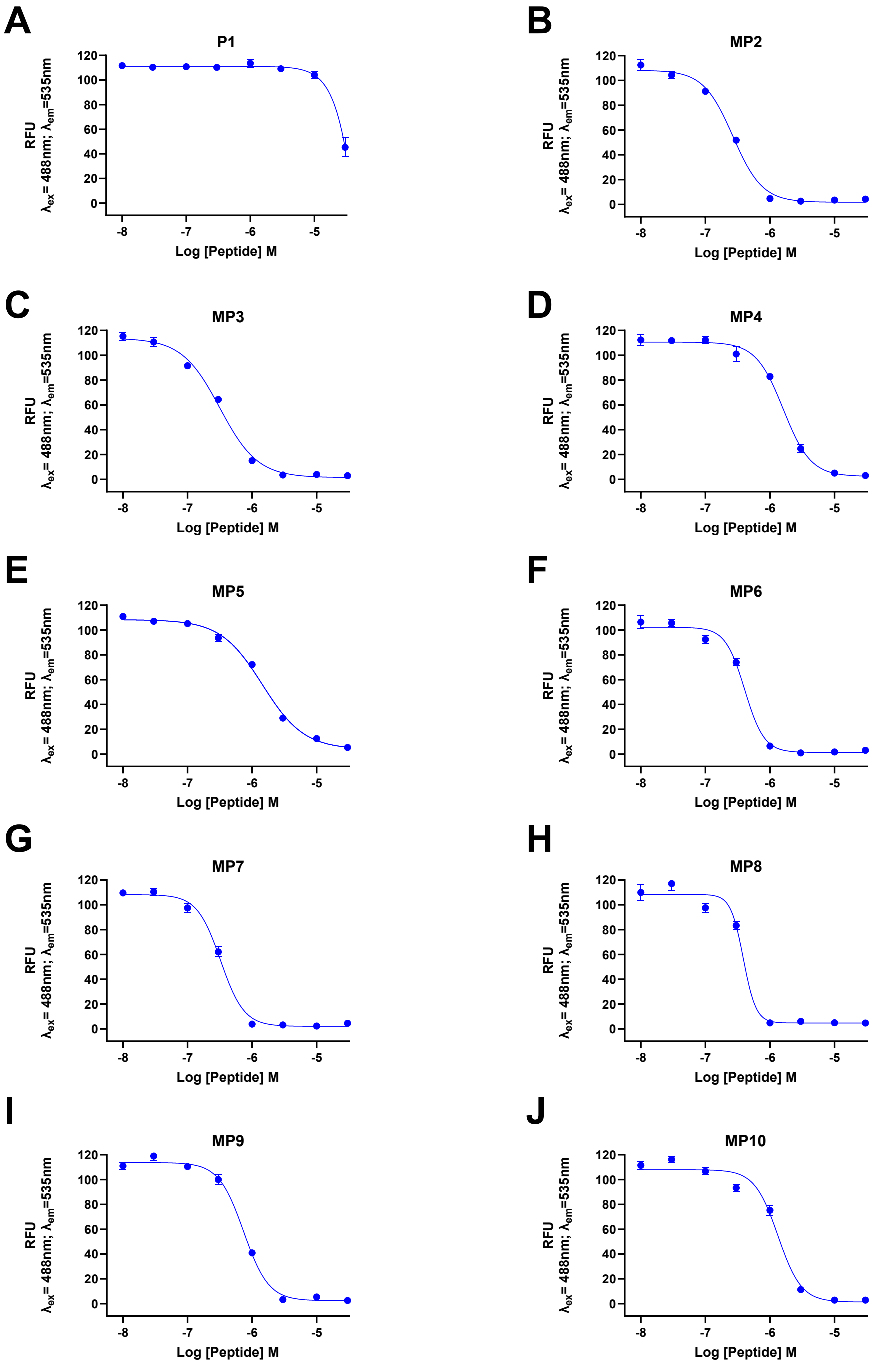

Figure S3. Dose-response curve of P1 and MP2-MP10 for the determination of K_D using guanine-induced fluorescence quenching. Each data point represents three independent replicates.

S5.3.2 Competition Binding Assay

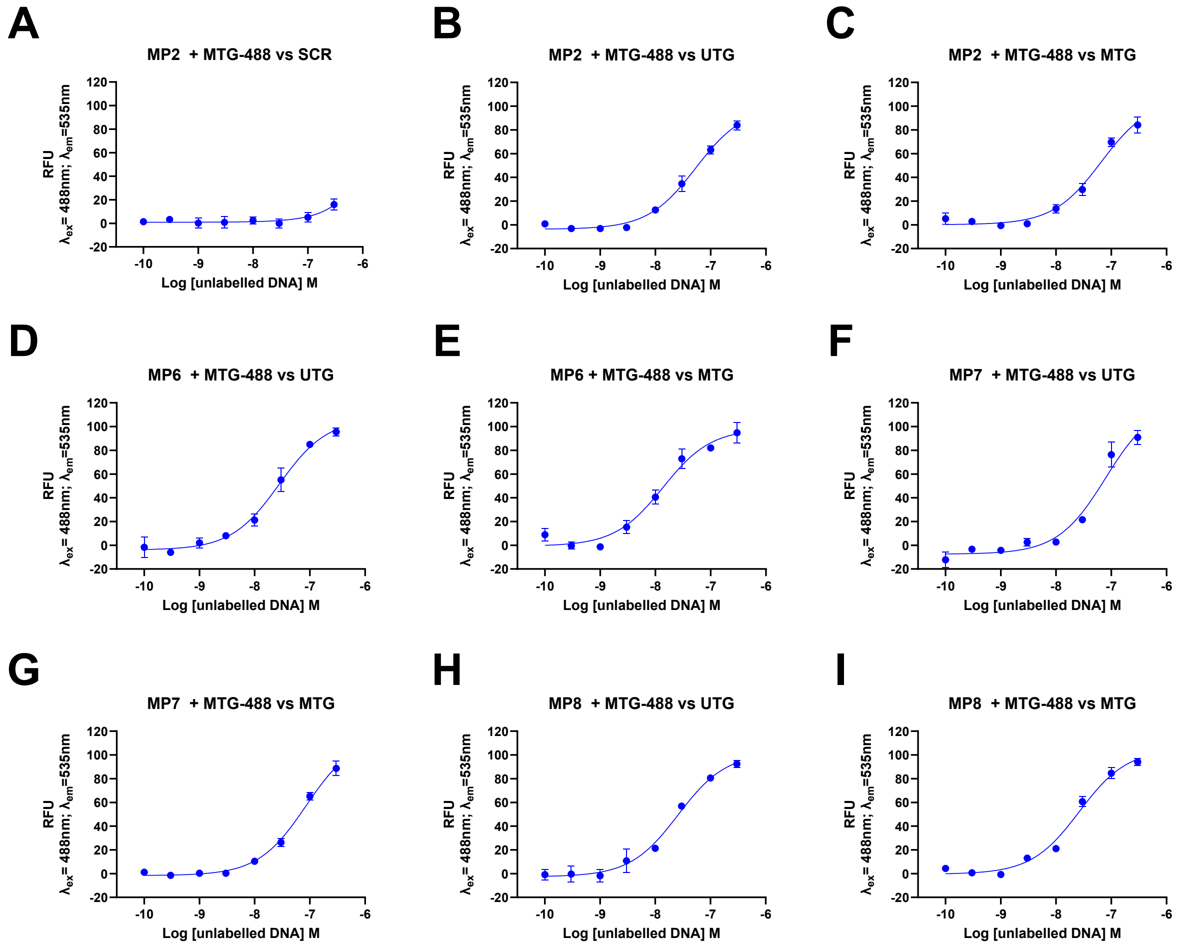

Figure S4. Competition ligand binding assays. **(A-C)** Dose-response curve of SCR, UTG and MTG unlabeled competitors against MP2 + MTG-488 complexes. **(D-E)** Dose-response curve of UTG and MTG unlabelled competitors against MP6 + MTG-488 complexes. **(F-G)** Dose-response curve of UTG and MTG unlabelled competitors against MP7 + MTG-488 complexes. **(H-I)** Dose-response curve of UTG and MTG unlabelled competitors against MP8+ MTG-488 complexes. IC_{50} was determined by titrating competitor DNA against metallopeptide (550 nM) and MTG-AF488 (10 nM) complexes. Each data point represents three independent replicates.

S6. Characterisation Data of Peptides and Stapled Metallopeptides

Table S1. Characterization data for linear peptides (P1-P10).

ID	Peptide Sequence	MW (g/mol)	Yield[a] (%)	RT[b] (min)	Purity[c] (%)	Molecular Formula	Mass[d]		
							Found	Calc.	Species
P1	Ac-SERQITIWFQNRRVKEKK-NH_2	2387	17	4.78	97	$C_{106}H_{175}N_{35}O_{28}$	796.4510	796.4521	$[M+3H]^{3+}$
P2	Ac-SERQITIWFQN**D**RVK**D**KK-NH_2	2332	8	4.94	99	$C_{103}H_{166}N_{32}O_{30}$	1166.6279	1166.6297	$[M+2H]^{2+}$
P3	Ac-SERQ**X**TIWFQN**D**RVK**D**KK-NH_2	2304	15	4.88	98	$C_{101}H_{162}N_{32}O_{30}$	1152.6125	1152.6140	$[M+2H]^{2+}$
P4	Ac-S**X**RQITIWFQN**D**RVK**D**KK-NH_2	2289	17	5.24	96	$C_{102}H_{166}N_{32}O_{28}$	573.0723	573.0719	$[M+4H]^{4+}$
P5	Ac-SERQITI**X**FQN**D**RVK**D**KK-NH_2	2231	16	4.78	87	$C_{96}H_{163}N_{31}O_{30}$	558.8131	558.8127	$[M+4H]^{4+}$
P6	Ac-SERQITIWFQN**D**R**L**K**D**KK-NH_2	2346	9	5.05	91	$C_{104}H_{168}N_{32}O_{30}$	782.7611	782.7608	$[M+3H]^{3+}$
P7	Ac-SERQITIWFQN**D**R**T**K**D**KK-NH_2	2334	6	4.96	96	$C_{102}H_{164}N_{32}O_{31}$	778.7496	778.7486	$[M+3H]^{3+}$
P8	Ac-SERQITIWFQN**D**R**F**K**D**KK-NH_2	2380	6	5.06	94	$C_{107}H_{166}N_{32}O_{30}$	794.0891	794.0889	$[M+3H]^{3+}$
P9	Ac-RQITIWFQN**D**RVK**D**-NH_2	1860	8	5.32	95	$C_{83}H_{130}N_{26}O_{23}$	620.6681	620.6674	$[M+3H]^{3+}$
P10	Ac-RQITIWFQN**D**RVK**D**KK-NH_2	2116	17	5.03	99	$C_{95}H_{154}N_{30}O_{25}$	1058.5924	1058.5902	$[M+2H]^{2+}$

Note: **X** = Aminoisobutyric acid; [a]Isolated yield; [b]LC-MS retention time obtained using mobile phase of MeCN/H_2O + 0.1% FA; [c]Purity by HPLC; [d]Data obtained on UHPLC Thermo Scientific LTQ orbitrap using mobile phase of MeCN/H_2O + 0.1% FA.

Table S2. Characterization data for stapled metallopeptides (MP2 – MP10).

ID	Peptide Sequence	MW (g/mol)	Yield[a] (%)	RT[b] (min)	Purity[c] (%)	Molecular Formula	Mass[d]		
							Found	Calc.	Species
MP2	Ac-SERQITIWFQN**D**RVK**D**KK-NH_2	2655	29	5.10	97	$C_{107}H_{170}N_{32}O_{34}Rh_2$	1327.5431	1327.5406	$[M+2H]^{2+}$
MP3	Ac-SERQ**X**TIWFQN**D**RVK**D**KK-NH_2	2626	31	5.02	99	$C_{105}H_{166}N_{32}O_{34}Rh_2$	1313.5215	1313.5250	$[M+2H]^{2+}$
MP4	Ac-S**X**RQITIWFQN**D**RVK**D**KK-NH_2	2610	35	5.37	98	$C_{106}H_{170}N_{32}O_{32}Rh_2$	1305.5405	1305.5457	$[M+2H]^{2+}$
MP5	Ac-SERQITI**X**FQN**D**RVK**D**KK-NH_2	2553	31	4.98	96	$C_{100}H_{167}N_{31}O_{34}Rh_2$	851.6852	851.6873	$[M+3H]^{3+}$
MP6	Ac-SERQITIWFQN**D**R**L**K**D**KK-NH_2	2668	35	5.13	99	$C_{108}H_{172}N_{32}O_{34}Rh_2$	1334.5498	1334.5485	$[M+2H]^{2+}$
MP7	Ac-SERQITIWFQN**D**R**T**K**D**KK-NH_2	2656	18	5.21	98	$C_{106}H_{168}N_{32}O_{35}Rh_2$	886.0190	886.0026	$[M+3H]^{3+}$
MP8	Ac-SERQITIWFQN**D**R**F**K**D**KK-NH_2	2702	26	5.23	98	$C_{111}H_{170}N_{32}O_{34}Rh_2$	901.6959	901.6973	$[M+3H]^{3+}$
MP9	Ac-RQITIWFQN**D**RVK**D**-NH_2	2181	31	5.58	96	$C_{87}H_{134}N_{26}O_{27}Rh_2$	2180.8076	2180.8095	$[M+H]^{+}$
MP10	Ac-RQITIWFQN**D**RVK**D**KK-NH_2	2438	30	5.25	98	$C_{99}H_{158}N_{30}O_{29}Rh_2$	813.3354	813.3380	$[M+3H]^{3+}$

Note: Underlined residue = Dirhodium metal staple site; **X** = Aminoisobutyric acid. [a]Isolated yield; [b]LCMS retention time obtained using mobile phase of MeCN/H_2O + 0.1% FA; [c]Purity by HPLC; [d]Data obtained on UHPLC Thermo Scientific LTQ orbitrap using mobile phase of MeCN/H_2O + 0.1% FA.

Table S3. CD Values for the determination of helicity, α-helicity ratio and fractional helicity (f_H) of linear peptides P1-P10 and metallopeptides MP2-MP10.

ID	Peptide Sequence	$[\theta_{222}]$ (nm)	$[\theta_{208}]$ (nm)	$\frac{\theta_{222}}{\theta_{208}}$	f_H (%)
**Linear Peptides**					
P1	Ac-SERQITIWFQNRRVKEKK-NH_2	-1481.53	-3358.02	0.44	7
P2	Ac-SERQITIWFQNDRVKDKK-NH_2	-2563.00	-5316.17	0.48	11
P3	Ac-SERQ**X**TIWFQNDRVKDKK-NH_2	-3330.77	-8897.65	0.38	13
P4	Ac-S**X**RQITIWFQNDRVKDKK-NH_2	-2105.39	-4742.39	0.44	9
P5	Ac-SERQITI**X**FQNDRVKDKK-NH2	-3243.32	-5617.77	0.58	13
P6	Ac-SERQITIWFQNDR**L**KDKK-NH_2	-2280.30	-5308.14	0.43	10
P7	Ac-SERQITIWFQNDR**T**KDKK-NH_2	-3055.41	-9826.75	0.31	12
P8	Ac-SERQITIWFQNDR**F**KDKK-NH_2	-3194.06	-9989.88	0.32	13
P9	Ac-RQITIWFQNDRVKD-NH_2	-6672.06	-10405.10	0.64	25
P10	Ac-RQITIWFQNDRVKDKK-NH_2	-1933.16	-4727.21	0.41	9
**Stapled Peptides**					
MP2	Ac-SERQITIWFQN**D**RVK**D**KK-NH_2	-3790.93	-3551.97	1.07	14
MP3	Ac-SERQ**X**TIWFQN**D**RVK**D**KK-NH_2	-5981.56	-3958.54	1.51	21
MP4	Ac-S**X**RQITIWFQN**D**RVK**D**KK-NH_2	-8146.46	-8074.14	1.01	28
MP5	Ac-SERQITI**X**FQN**D**RVK**D**KK-NH_2	-6788.96	-7118.73	0.95	24
MP6	Ac-SERQITIWFQN**D**R**L**K**D**KK-NH_2	-6046.18	-6195.43	0.98	21
MP7	Ac-SERQITIWFQN**D**R**T**K**D**KK-NH_2	-3844.02	-3766.65	1.02	15
MP8	Ac-SERQITIWFQN**D**R**F**K**D**KK-NH_2	-5550.51	-5370.15	1.03	20
MP9	Ac-RQITIWFQN**D**RVK**D**-NH_2	-6739.98	-7436.14	0.91	25
MP10	Ac-RQITIWFQN**D**RVK**D**KK-NH_2	-6335.99	-7568.42	0.84	23

Note: Underlined residue = Dirhodium metal staple site; **X** = Aminoisobutyric acid; f_H = fraction helicity; [θ] = mean residual ellipticity in deg cm^2 $dmol^{-1}residue^{-1}$.

S7. Key interactions of HOXB13 with primary DNA binding motif

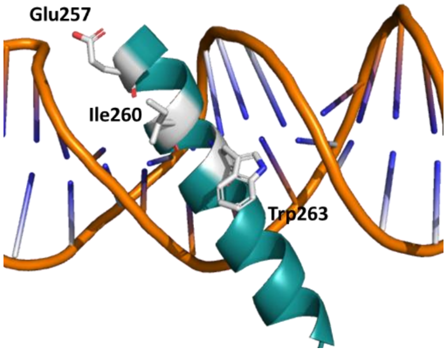

Figure S5. Crystal structure of HOXB13 complexed with its cognate methylated DNA motif (PDB ID = 5ef6). Ile[260] is solvent-exposed; Glu[257] or Trp[263] are positioned in proximity to the DNA backbones. Molecular structures were visualized and figures prepared using PyMOL.[6]

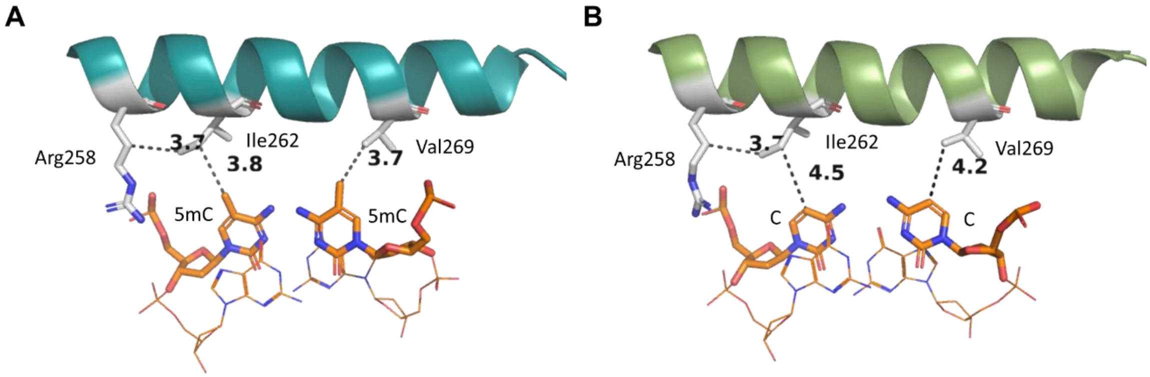

Figure S6. (**A**) Crystal structure of HOXB13 complexed with its cognate methylated DNA motif (PDB ID = 5ef6). Methyl-methyl contact was observed for 5-methylcytosine residues with Ile[262] and Val[269]. (**B**) Crystal structure of HOXB13 complexed with its cognate unmethylated DNA motif (PDB ID = 5edn). Methyl-methyl contact was absent. Molecular structures were visualized and figures prepared using PyMOL.[6]

S8. ^{1}H NMR and HPLC data

$Rh_2(OAc)_2(TFA)_2$.MeCN (1)[1]

^{1}H NMR (400 MHz) in $CDCl_3$

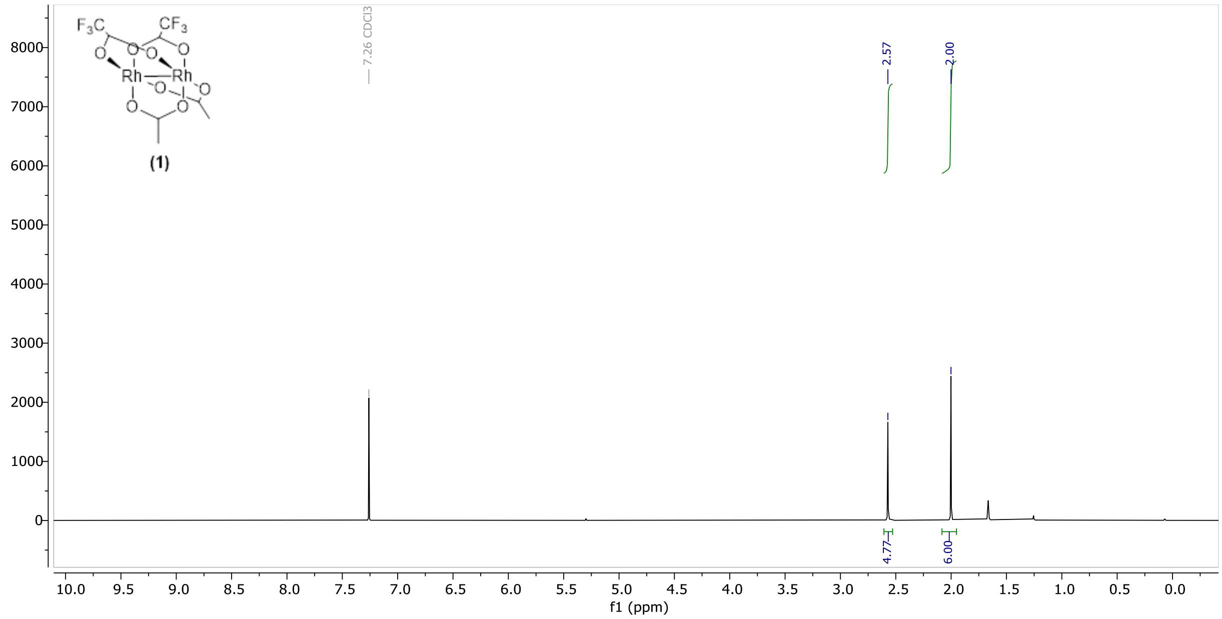

^{19}F NMR (376 MHz) in $CDCl_3$

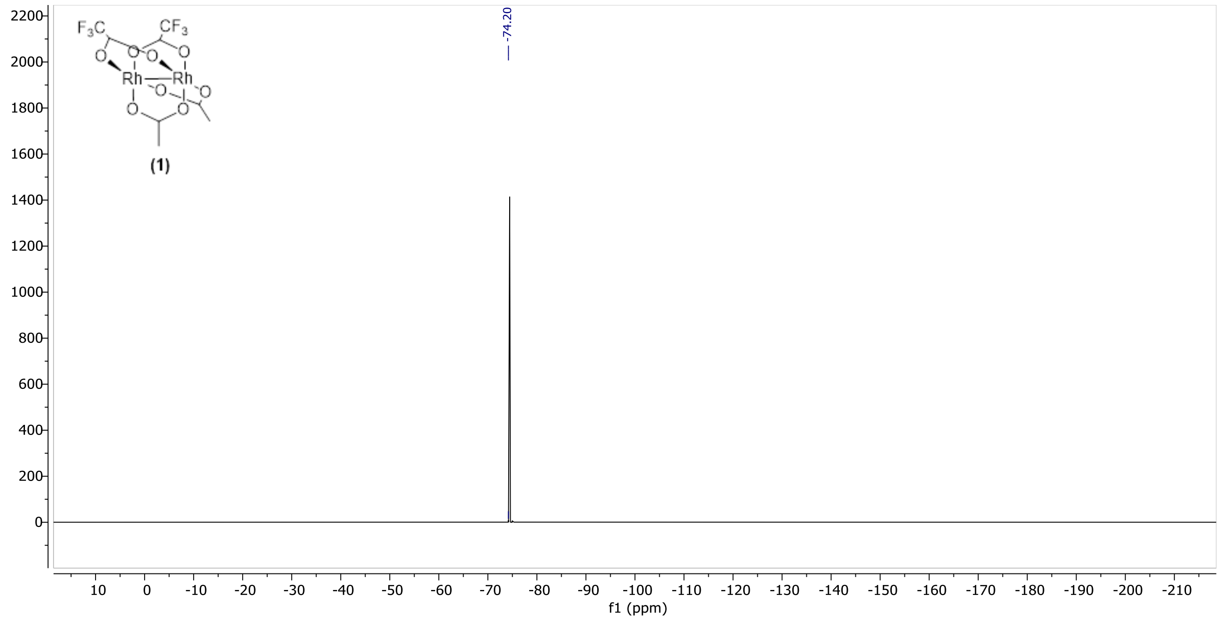

α3-parent peptide (P1)

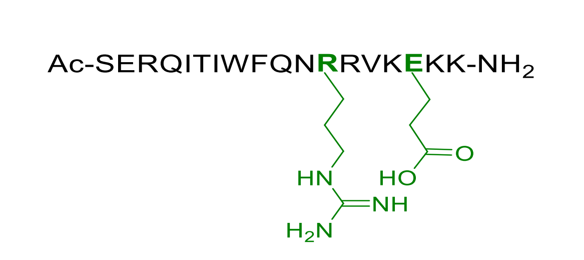

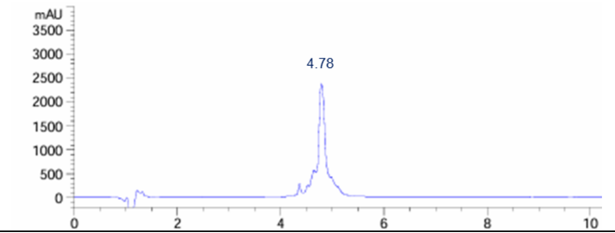

^1H NMR (600 MHz) in 9:1 H_2O:D_2O

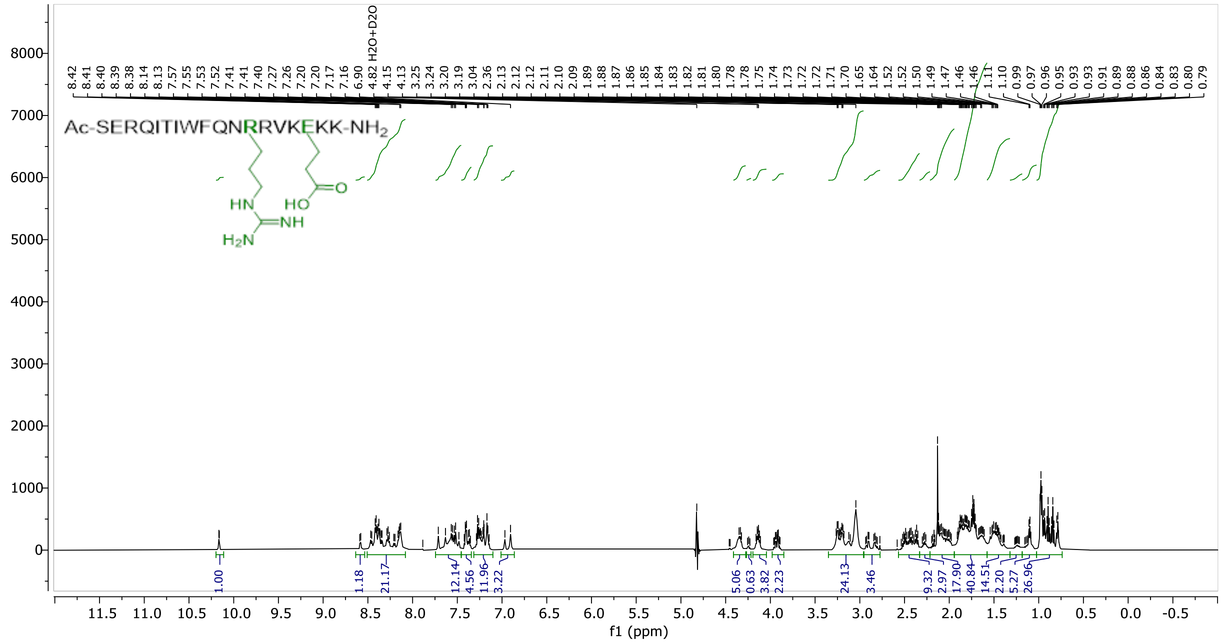

Ac-SERQITIWFQNDRVKDKK-NH$_2$ (MP2)

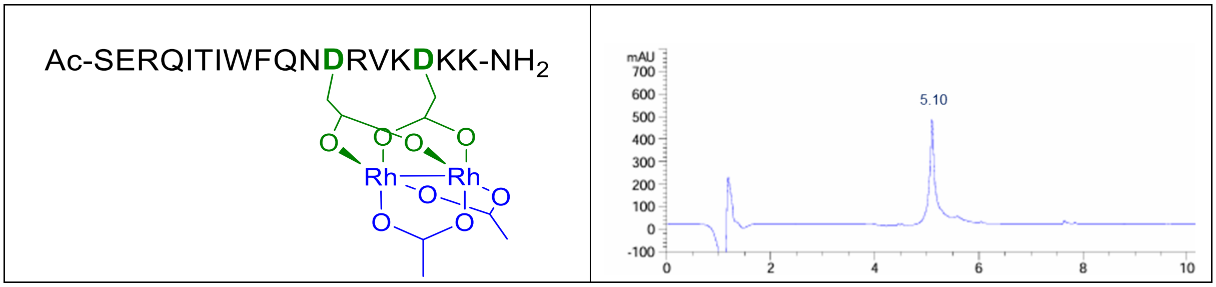

^{1}H NMR (500 MHz) in 9:1 H_2O:D_2O

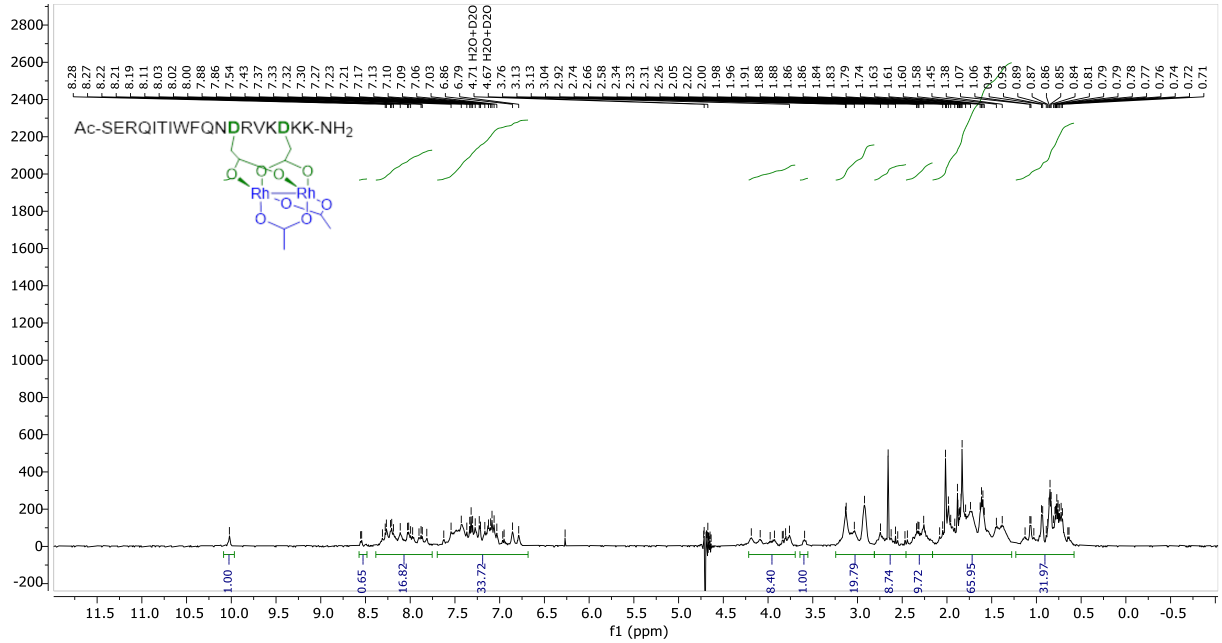

Ac-SERQXTIWFQN<u>D</u>RVK<u>D</u>KK-NH2 (MP3)

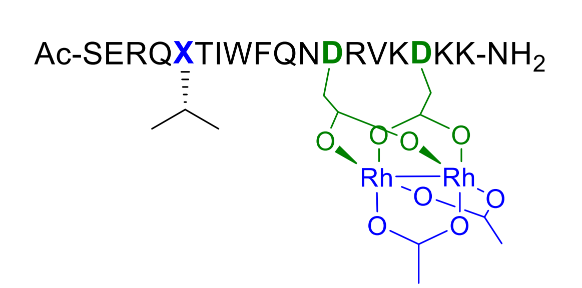

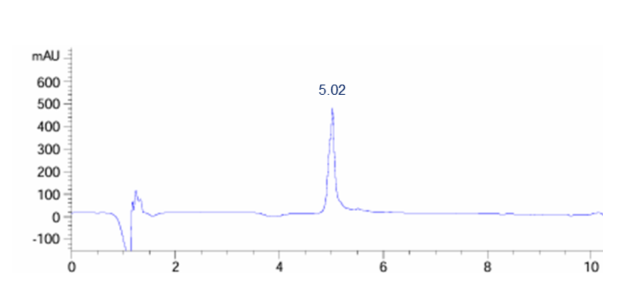

^{1}H NMR (500 MHz) in 9:1 H_2O:D_2O

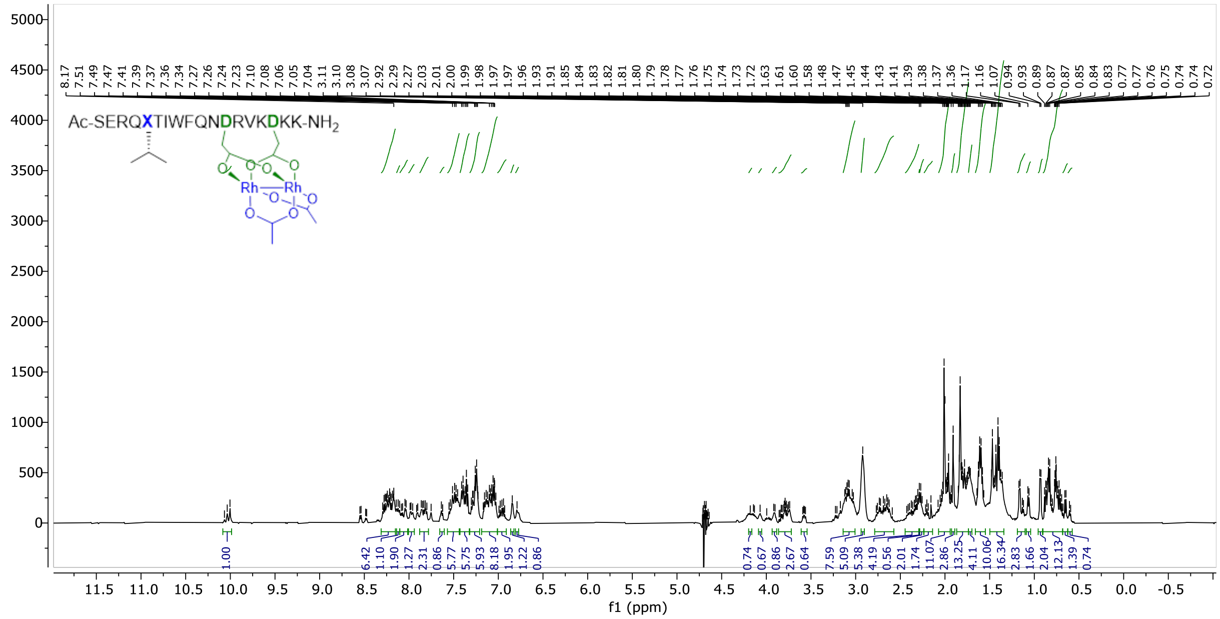

Ac-SXRQITIWFQN<u>D</u>RVK<u>D</u>KK-NH_2 (MP4)

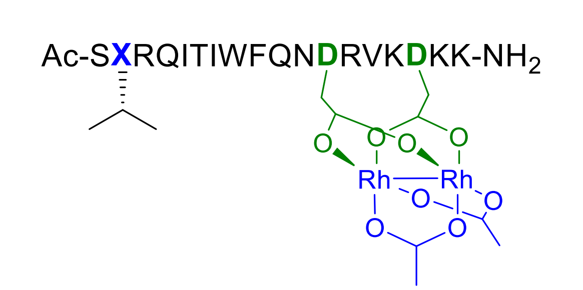

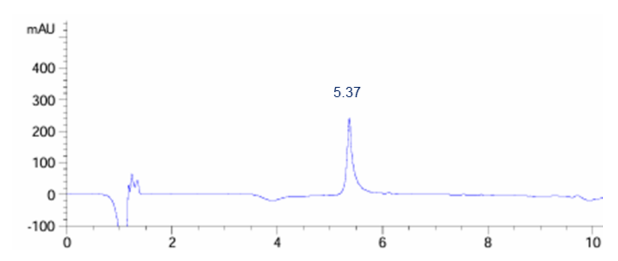

^{1}H NMR (500 MHz) in 9:1 H_2O:D_2O

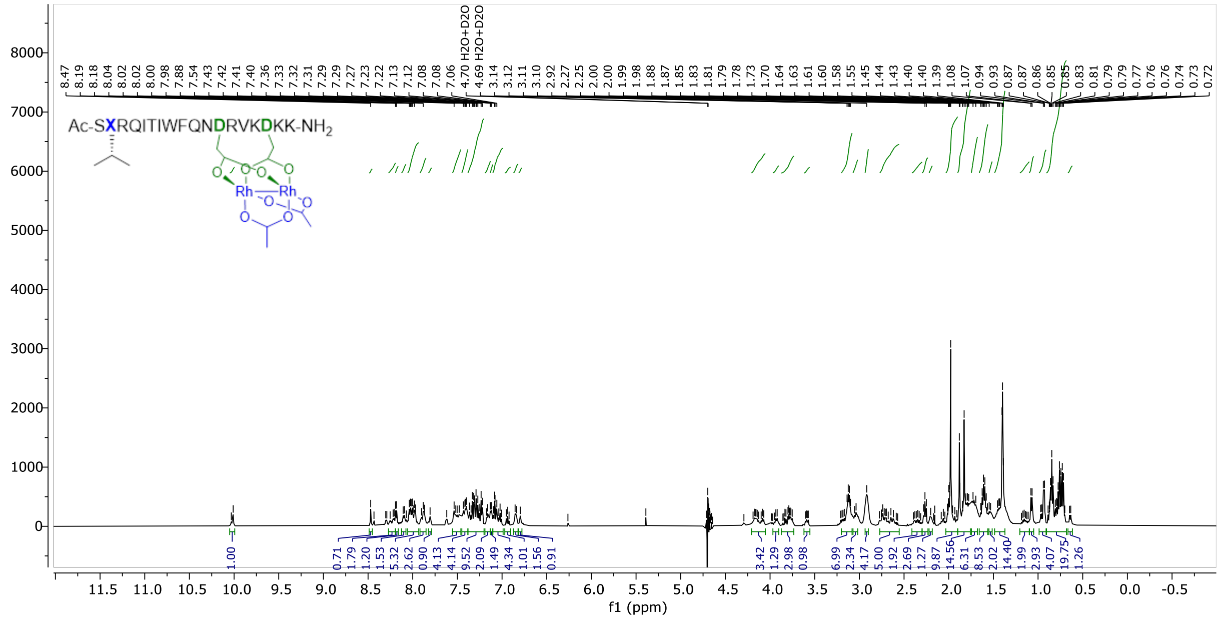

Ac-SERQITIXFQN<u>D</u>RVK<u>D</u>KK-NH$_2$ (MP5)

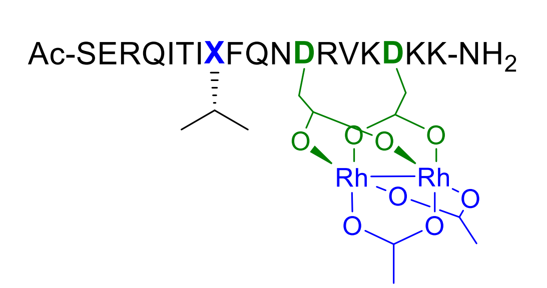

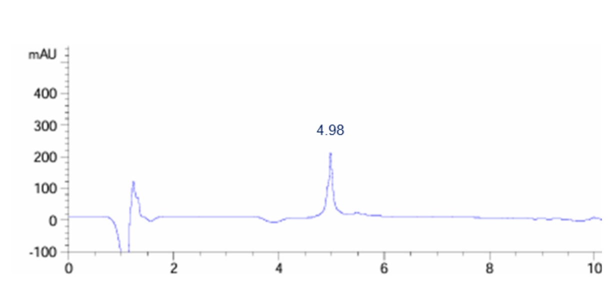

^{1}H NMR (500 MHz) in 9:1 H_2O:D_2O

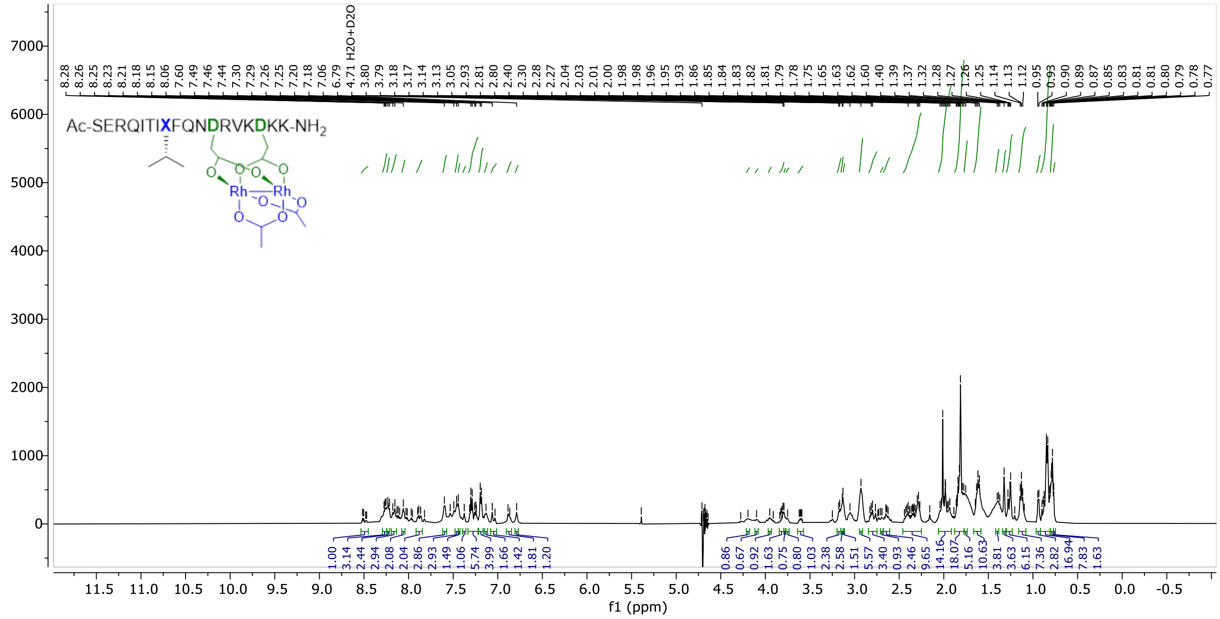

Ac-SERQITIWFQN<u>D</u>RLK<u>D</u>KK-NH₂ (MP6)

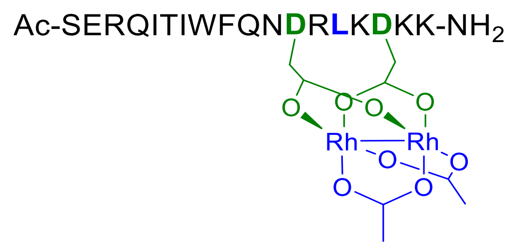

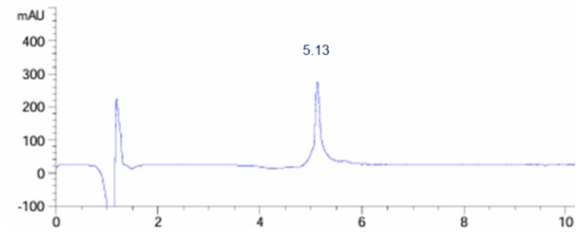

5.2

^{1}H NMR (500 MHz) in 9:1 H_2O:D_2O

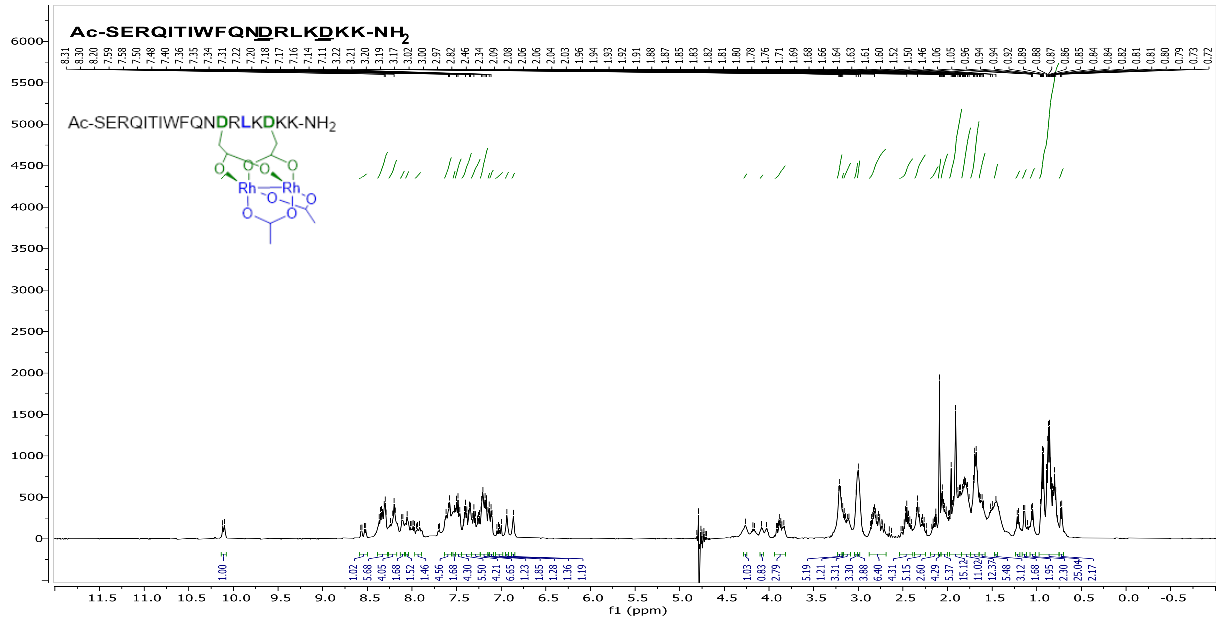

Ac-SERQITIWFQN<u>D</u>RTK<u>D</u>KK-NH₂ (MP7)

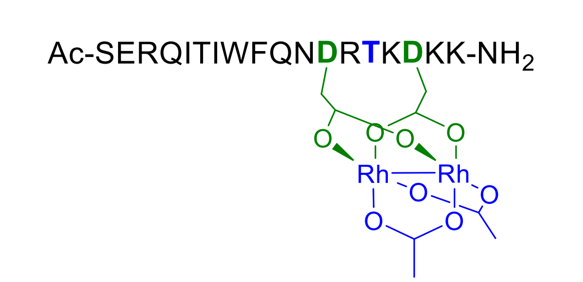

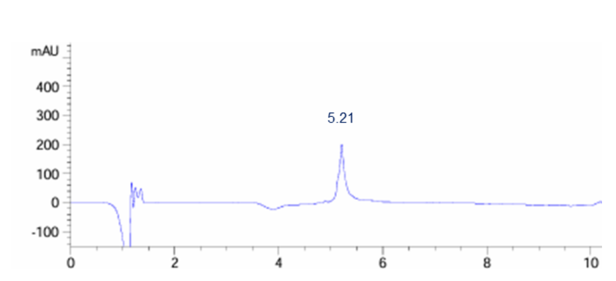

^{1}H NMR (500 MHz) in 9:1 H_2O:D_2O

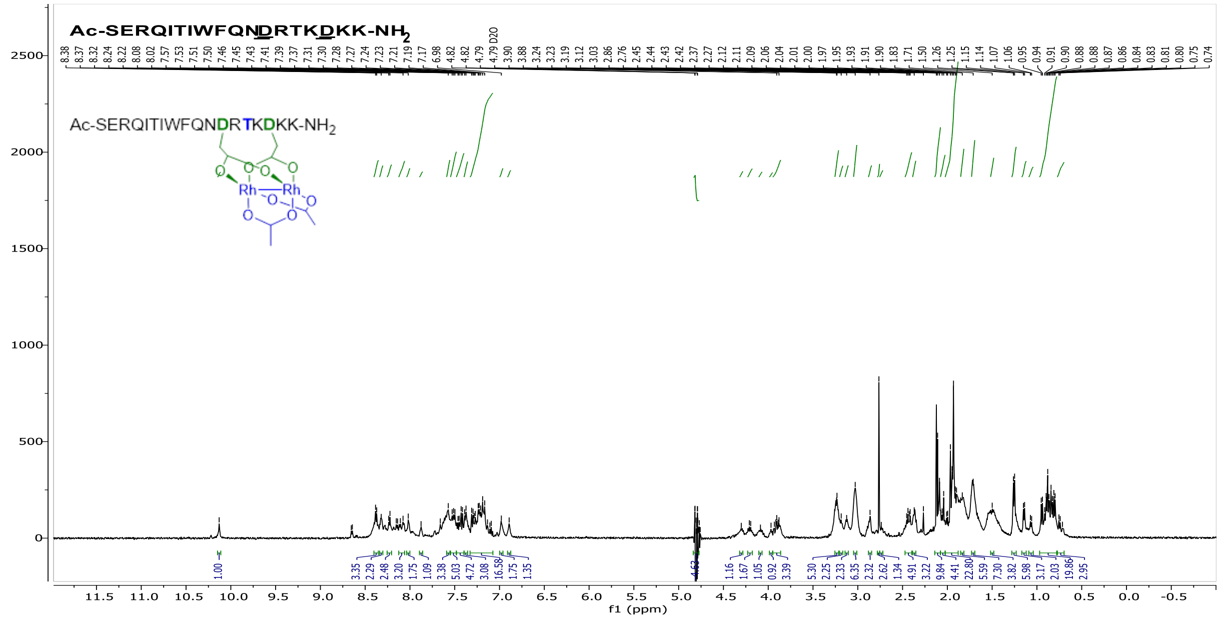

Ac-SERQITIWFQN$\underline{D}$RFK$\underline{D}$KK-NH$_2$ (MP8)

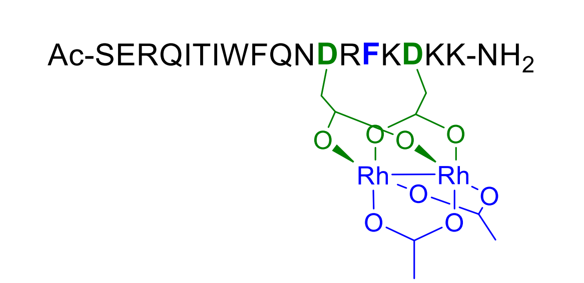

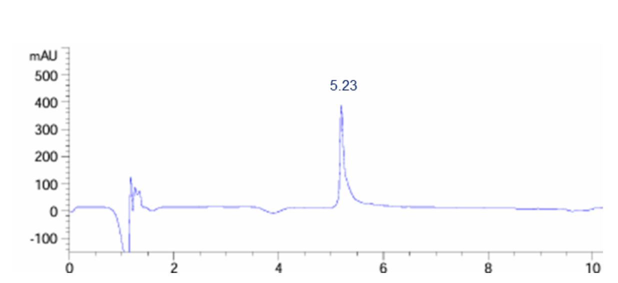

^{1}H NMR (500 MHz) in 9:1 H_2O:D_2O

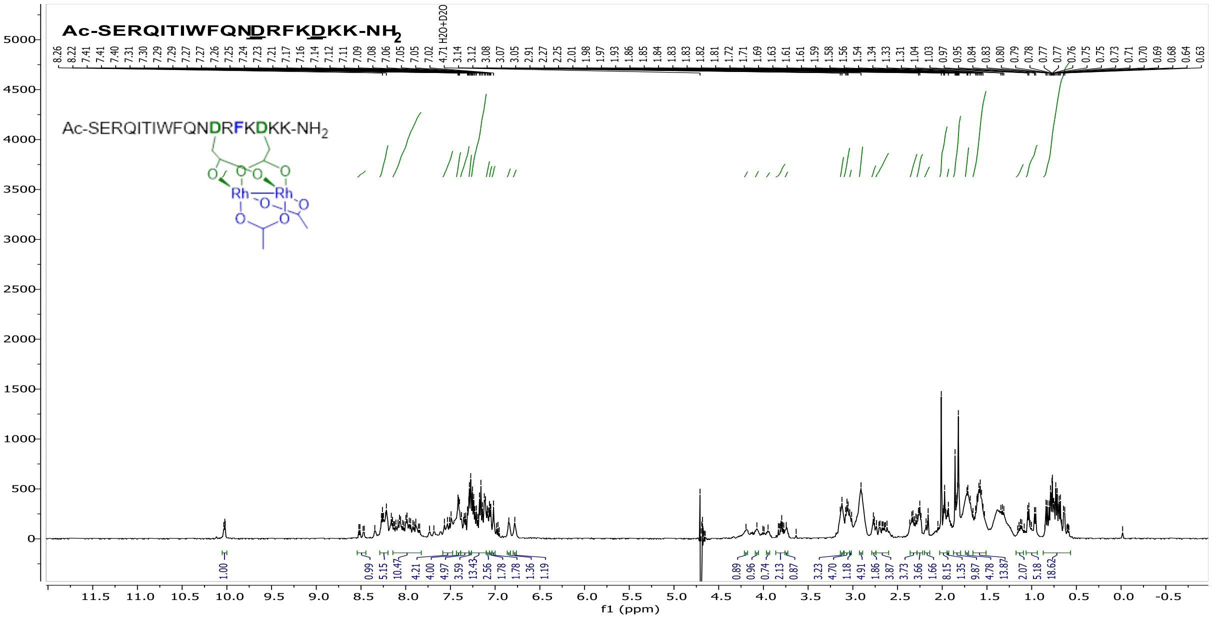

Ac-RQITIWFQN<u>D</u>RVK<u>D</u>-NH$_2$ (MP9)

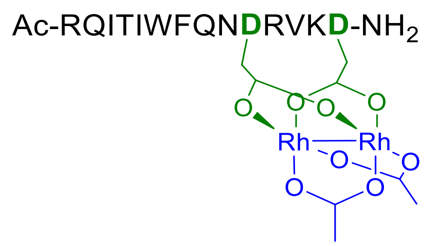

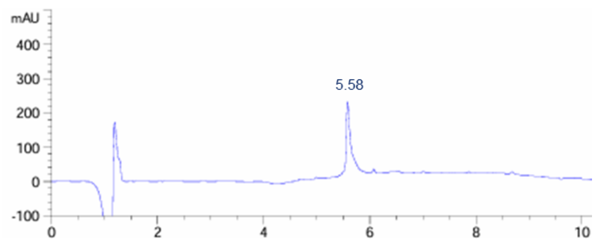

^{1}H NMR (500 MHz) in 9:1 H_2O:D_2O

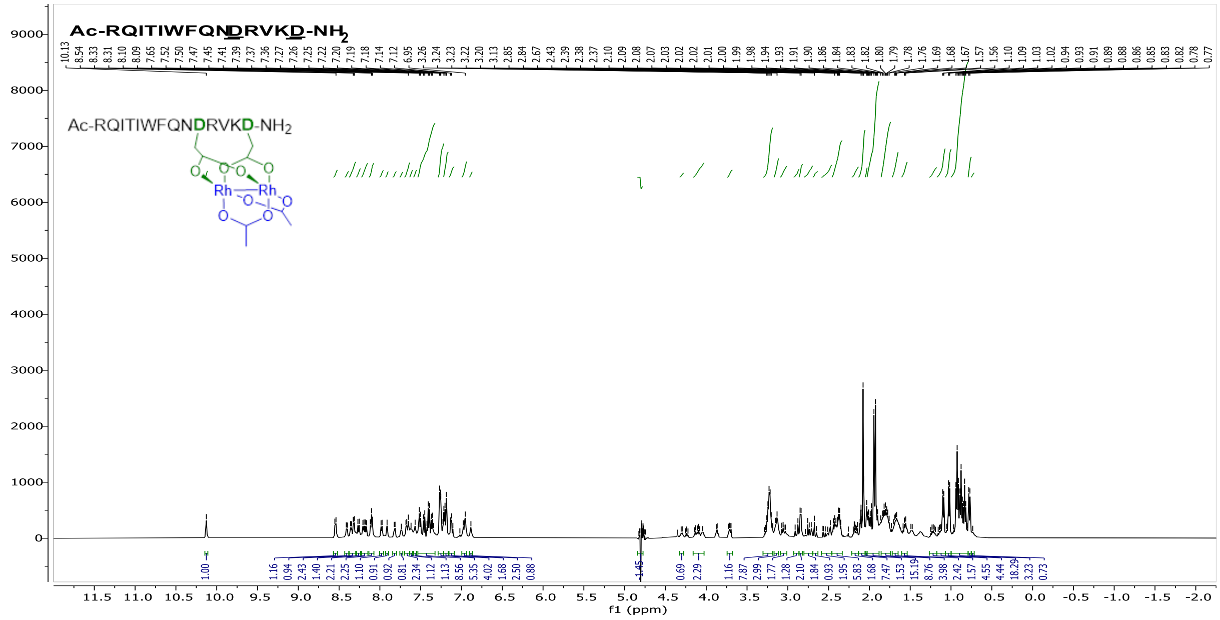

Ac-RQITIWFQN<u>D</u>RVK<u>D</u>KK-NH$_2$ (MP10)

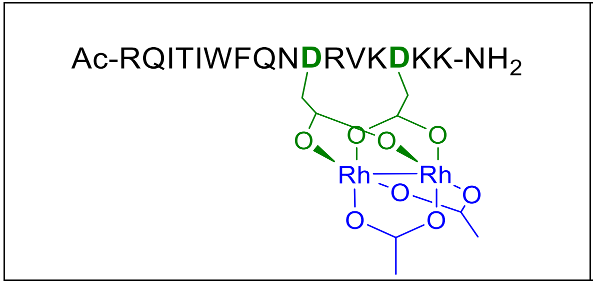

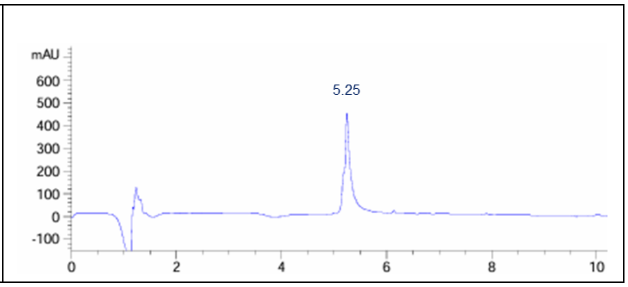

^{1}H NMR (500 MHz) in 9:1 H_2O:D_2O

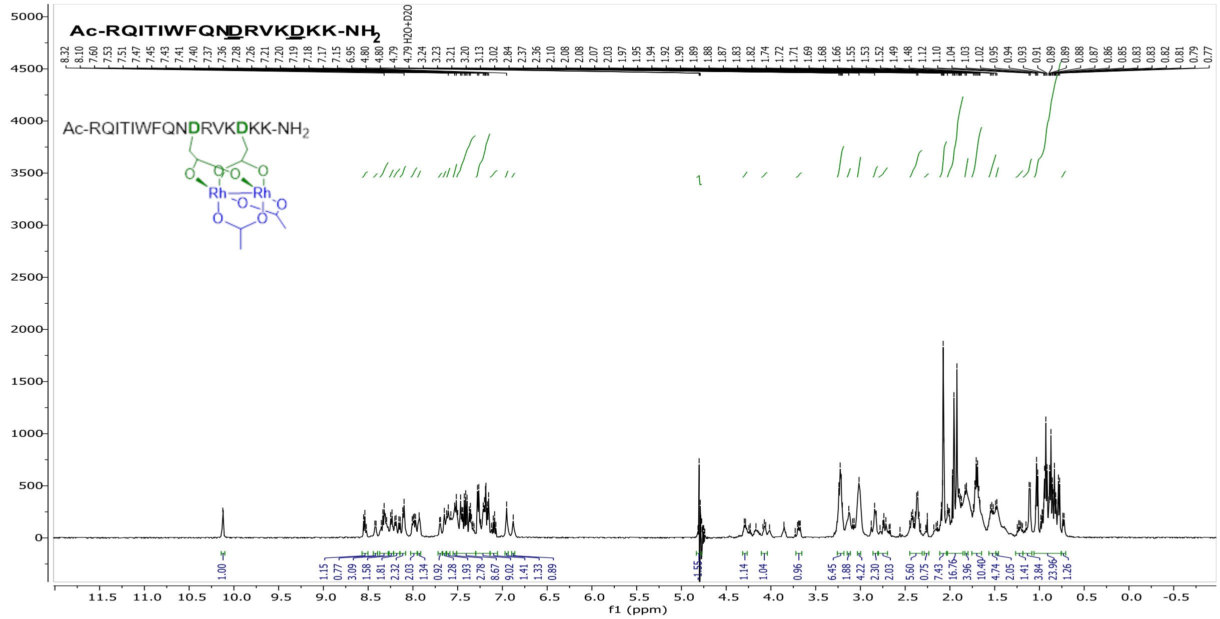

S9. Proteolytic stability assay

Stapled peptide **MP2** and linear parent peptide **P1** were subjected to trypsin digestion to assess their respective proteolytic stability. 200 µM of peptide samples and 0.5 µM of trypsin (from porcine pancreas; Sigma-Aldrich) were incubated in 10 mM ammonium carbonate buffer at pH 8.2 and 25°C for 2 h. 20 µL aliquots were removed at 0 h, 5 min, 10 min, 20 min, 30 min, 60 min and 120 min timepoints. 20 µL of quenching cocktail (3% TFA in H_2O) was added to inhibit trypsin activity prior to LC-MS analysis to monitor proteolytic degradation.

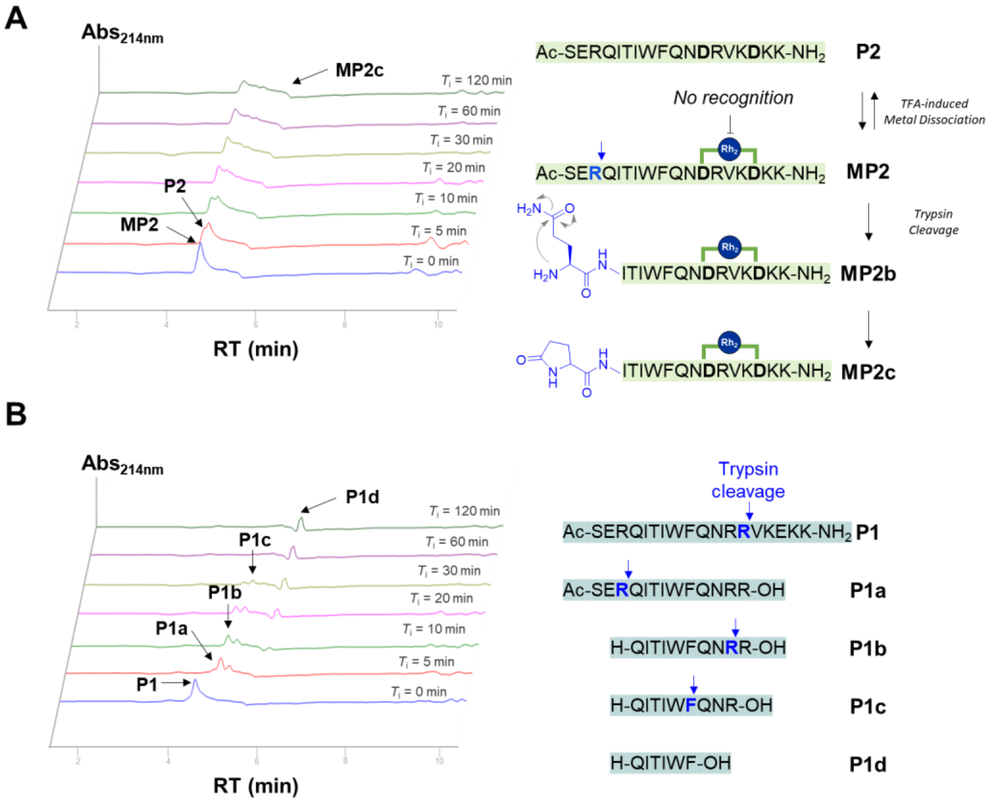

Figure S7. LC-MS analysis of peptide proteolysis by trypsin at 25°C over a period of 120 min incubation time (T_i). (**A**) Proteolysis of MP2. TFA-induced metal dissociation from MP2 to form P2 was observed in the presence of excess TFA used during quenching. No cleavage was observed at the metal-stapled site. Minor trypsin cleavage products at a N-terminal site were observed. (**B**) Proteolysis of P1. Multiple products of trypsin cleavage observed, as denoted by their respective retention time (RT). Atypical cleavage of Phe-Gln site led to the release of hydrophobic peptide **P1d** observed at a higher retention time (RT).

S10. References

(1) Lou, Y.; Remarchuk, T. P.; Corey, E. J. Catalysis of Enantioselective [2+1]-Cycloaddition Reactions of Ethyl Diazoacetate and Terminal Acetylenes Using Mixed-Ligand Complexes of the Series Rh2(RCO2)n (L*4-n). Stereochemical Heuristics for Ligand Exchange and Catalyst Synthesis. *J. Am. Chem. Soc.* **2005**, *127* (41), 14223–14230. https://doi.org/10.1021/ja052254w.

(2) Zaykov, A. N.; MacKenzie, K. R.; Ball, Z. T. Controlling Peptide Structure with Coordination Chemistry: Robust and Reversible Peptide–Dirhodium Ligation. *Chemistry – A European Journal* **2009**, *15* (36), 8961–8965. https://doi.org/10.1002/chem.200901266.

(3) Dreier, L.; Wider, G. Concentration Measurements by PULCON Using X-Filtered or 2D NMR Spectra. *Magnetic Resonance in Chemistry* **2006**, *44* (S1), S206–S212. https://doi.org/10.1002/mrc.1838.

(4) Luo, P.; Baldwin, R. L. Mechanism of Helix Induction by Trifluoroethanol: A Framework for Extrapolating the Helix-Forming Properties of Peptides from Trifluoroethanol/Water Mixtures Back to Water. *Biochemistry* **1997**, *36* (27), 8413–8421. https://doi.org/10.1021/bi9707133.

(5) de Araujo, A. D.; Hoang, H. N.; Kok, W. M.; Diness, F.; Gupta, P.; Hill, T. A.; Driver, R. W.; Price, D. A.; Liras, S.; Fairlie, D. P. Comparative α-Helicity of Cyclic Pentapeptides in Water. *Angewandte Chemie International Edition* **2014**, *53* (27), 6965–6969. https://doi.org/10.1002/anie.201310245.

(6) The PyMOL Molecular Graphics System, Version 3.0 Schrödinger, LLC.
